# Nutrition and diet in rheumatoid arthritis, axial spondyloarthritis, and psoriatic arthritis: a systematic review

**DOI:** 10.3389/fmed.2025.1655165

**Published:** 2025-09-15

**Authors:** Kaat Van den Bruel, Myroslava Kulyk, Barbara Neerinckx, Kurt De Vlam

**Affiliations:** ^1^Department of Rheumatology, University Hospitals Leuven, Leuven, Belgium; ^2^Skeletal Biology and Engineering Research Center, Department of Development and Regeneration, Leuven, Belgium

**Keywords:** rheumatoid arthritis, psoriatic arthritis, spondyloarthritis, nutrition, diet, probiotics, synbiotics, polyunsaturated fatty acids (PUFA)

## Abstract

**Introduction:**

This systematic review aimed to evaluate the effects of specific diets, dietary supplements, and probiotics on disease activity, inflammation, and immune response in patients with rheumatoid arthritis (RA), axial spondyloarthritis (axSpA), and psoriatic arthritis (PsA).

**Methods:**

A systematic literature search was conducted in PubMed, Embase, and the Cochrane Library. Randomized clinical trials (RCTs) of patients with RA, axSpA, or PsA undergoing dietary or nutritional interventions were included. Duplicates were removed using EndNote and Rayyan, and study quality was assessed with the Academy of Nutrition and Dietetics Quality Criteria Checklist for Primary Research. Outcomes of interest were changes in immune response, inflammatory biomarkers, and disease activity.

**Results:**

From 2,250 screened articles, 49 studies met the inclusion criteria. In RA, vegan, anti-inflammatory, and Mediterranean diets improved disease activity, inflammation markers, and quality of life. For axSpA, evidence was limited, though supplementation with polyunsaturated fatty acids (PUFAs) showed potential benefits. Across conditions, nutritional supplements such as PUFAs, vitamin D, pomegranate extract, and ginger demonstrated anti-inflammatory and immunomodulatory effects. Probiotics and synbiotics had variable impacts, with synbiotics reducing interleukin-17 (IL-17) levels. In PsA, a hypocaloric diet supplemented with omega-3 fatty acids was associated with reduced disease activity.

**Discussion:**

Dietary interventions and supplementation may support the management of chronic arthritis through modulation of inflammatory and immune pathways. However, due to heterogeneity in study designs, interventions, and outcomes, a meta-analysis was not feasible, and results were synthesized narratively. While findings suggest potential benefits as adjuncts to pharmacological treatment, further high-quality RCTs are required to confirm long-term clinical efficacy.

**Systematic review registration:**

The systematic review is registered in PROSPERO under ID CRD420251010982. https://www.crd.york.ac.uk/PROSPERO/view/CRD420251010982.

## 1 Introduction

The global prevalence of chronic arthritis is increasing worldwide (1). While pharmacological treatments are well-established, the role of diet and nutrition in disease management remains underrecognized. Chronic arthritis, including rheumatoid arthritis (RA), psoriatic arthritis (PsA), and axial spondyloarthritis (axSpA), has a complex pathogenesis involving genetic predisposition, environmental factors, and immune system activation (2, 3). Diet can modulate the immune response by influencing gut microbiota composition, regulating inflammatory pathways, and altering the balance between pro-inflammatory and anti-inflammatory cytokines (3). Various dietary factors, such as processed foods and additives, can interfere with nutrient absorption, leading to anti-nutritional effects (4). Conversely, optimal nutrition may reduce or delay immune-mediated chronic diseases (5).

Anti-inflammatory diets are dietary patterns designed to reduce chronic inflammation by emphasizing the consumption of foods with anti-inflammatory properties, such as fruits, vegetables, whole grains, nuts, seeds, and fatty fish, while minimizing pro-inflammatory foods like processed foods, added sugars, and red meats (6). According to the systematic review by Genel et al. (7) the anti-inflammatory diet is hypothesized to alleviate symptoms of inflammatory conditions such as osteoarthritis and RA by reducing levels of inflammatory biomarkers, particularly C-reactive protein (CRP) and interleukin-6 (IL-6).

The Mediterranean diet (MD) is a well-researched dietary pattern characterized by high consumption of extra-virgin olive oil (EVOO), fruits, vegetables, nuts, legumes, whole grains, and moderate intake of fish and wine, which collectively contribute to its anti-inflammatory properties. EVOO, rich in polyphenols such as oleuropein and hydroxytyrosol, plays a key role in reducing oxidative stress, low-density lipoprotein (LDL) oxidation, and inflammatory markers (8). Additionally, the MD positively influences gut microbiota composition, enhancing beneficial microbial populations that regulate immune responses and inflammatory pathways (9). Weight loss interventions and dietary regimens, such as gluten-free and Mediterranean diets or supplement use, may potentially improve the natural progression of chronic arthritis and its response to therapy (10). The variation in prevalence of chronic arthritis between continents, with higher rates observed in Western countries, might be indicative (2, 11).

Fatty acids serve as important macronutrients for immunomodulation, with n-3 polyunsaturated fatty acids demonstrating particularly beneficial effects, such as reducing inflammation, modulating immune cell function, and supporting overall immune system balance (5). Polyunsaturated fatty acids (PUFA) (12) can be classified into omega-3 (n-3) and omega-6 fatty acids based on the location of the first double bond relative to the methyl end of the fatty acid chain. The group of n-3 PUFA includes alpha-linolenic acid (ALA), eicosapentaenoic acid (EPA), and docosahexaenoic acid (DHA), which are found in fatty fish (e.g., salmon, mackerel, sardines), as well as in plant-based sources like flaxseeds, chia seeds, and walnuts. The group of n-6 PUFA includes linoleic acid (LA) and arachidonic acid (AA), which are predominantly present in vegetable oils such as soybean, corn, and sunflower oil (12).

Monounsaturated fatty acids (MUFA) (13) are regarded as beneficial fats and include omega-9 fatty acids. The body is able to create omega-9 fatty acids on its own, in contrast to n-3 PUFA and omega-6 fatty acids, which are regarded as necessary fatty acids. Consuming foods high in omega-9 can still be advantageous for general health. The most prevalent omega-9 fatty acid is oleic acid, which may be found in large amounts in foods like avocados and almonds as well as in olive oil. Additionally, polyphenols and carotenoids are promising antioxidants in the context of rheumatic diseases (10).

Flaxseed, derived from the plant Linumusitatissimum, is recognized for its potential health benefits, particularly in managing inflammatory conditions. It is rich in alpha-linolenic acid (ALA), an n-3 PUFA fatty acid known for its anti-inflammatory properties (14). A meta-analysis (15) of 32 clinical trials examined the impact of flaxseed-derived products on inflammatory biomarkers. The analysis revealed that flaxseed intake significantly reduced levels of high-sensitivity CRP (hs-CRP) and TNF-α, both of which are markers of inflammation. However, no significant changes were observed in IL-6 and standard CRP levels (10).

Probiotics, prebiotics, and synbiotics affect the immune system, inflammatory biomarkers, and disease activity (16). Certain meals, such as yogurt, kefir, and other fermented foods, as well as supplements, contain probiotics. Bifidobacterium and Lactobacillus species are common probiotic bacteria. Prebiotics are indigestible fibers that feed beneficial bacteria that are already in the stomach. In the gut, they basically serve as fertilizer for probiotics and other good bacteria. Synbiotics are a combination of probiotics and prebiotics that include good bacteria along with substances that help them grow. Probiotics modulate both innate and adaptive immune responses by influencing the activities of dendritic cells, macrophages, and T and B lymphocytes. Toll-like receptor activation is a key mechanism through which probiotics exert their immunomodulatory effects (17). According to several studies (16), prebiotics can affect immunological and metabolic parameters like IL-6, insulin resistance, and blood glucose levels (18). These findings suggest that the gut microbiota has a role in maintaining the host’s health by controlling the host’s immunological response and metabolism in response to diet (18). While prebiotics predominantly impact the large intestine, probiotics primarily affect both the small and large intestines, therefore combining the two, known as synbiotics, may have a synergistic effect (19).

Emerging evidence highlights the role of gut microbiota in modulating immune responses and influencing the onset and progression of RA (20). Dysbiosis, or an imbalance in gut microbial composition, has been associated with increased intestinal permeability, systemic inflammation, and heightened immune activation (21). Among the most studied probiotics, *Lactobacillus casei* and *Lactobacillus acidophilus* have demonstrated anti-inflammatory effects and improvements in arthritis severity in preclinical and clinical studies (22, 23). For instance, animal studies revealed that supplementation with these strains reduced pro-inflammatory cytokines such as IL-6 and TNF-α, while increasing anti-inflammatory mediators like IL-10 (22).

This systematic review aims to synthesize existing evidence on the impact of dietary patterns, nutritional supplements, and probiotics on disease activity, inflammation, and immune modulation in patients with RA, axSpA, and PsA.

## 2 Methods

### 2.1 Search strategy

A systematic literature search was conducted from inception to December 2024 in three different electronic databases: PubMed, Embase, and the Cochrane Library. Predefined search terms were used focusing on two key concepts: chronic arthritis and nutrition/diet. Included search terms were “Psoriatic Arthritis,” “Spondyloarthritis,” and “Rheumatoid Arthritis,” incorporating both MeSHterms (e.g., “Arthritis, Psoriatic”[Mesh], “Spondyloarthritis” [Mesh], “Arthritis, Rheumatoid” [Mesh]) and free-text keywords (e.g., “Psoriatic Arthropathy,” “Spinal Arthritis”). For nutrition and diet, included search terms were “Dietary Supplements,” “Probiotics,” “Mediterranean Diet,” and “Nutrition Therapy,” with both MeSH terms (e.g., “Dietary Supplements”[Mesh], “Diet, Mediterranean” [Mesh]) and text words (e.g., “Herbal Supplement,” “Food Supplement,” “Medical Nutrition Therapy”). The Boolean logic (#1 AND #2) was used to combine the two concepts, ensuring specificity. Filters were applied in Embase to exclude conference abstracts. This search was limited to published peer-reviewed articles and did not include grey literature or trial registries.

### 2.2 Inclusion and exclusion criteria

The study selection process involved the use of Rayyan software for screening. Randomized controlled trials (RCTs) recruiting participants with a diagnosis of RA, axSpA or PsA and a minimum age of 18 years, were eligible for inclusion in the study. Trials comparing a specific dietary intervention (e.g., a particular diet, vitamins, or probiotics) with a control or alternative intervention group were included. Studies that met the eligibility requirements had to compare the effects of the intervention to either the control group, which received no intervention, or the comparison group, which received another type of intervention. Studies published in languages other than English were excluded due to the inability to ensure accurate interpretation. Research involving experimental animal models, trials without a control group, observational studies conducted retrospectively, and studies lacking information on disease outcomes or other critical factors for disease activity were also excluded.

### 2.3 Data extraction

The systematic review was performed according to the Preferred Reporting Items for Systematic Review and Meta-Analysis (PRISMA) statement (24). Data extraction included study design, participant characteristics, intervention type, and key outcomes. Information on funding sources and potential conflicts of interest was also recorded to evaluate bias. After removing duplicate records, titles and abstracts were screened for relevance. Studies without full text available were removed, followed by a selection process based on the inclusion and exclusion criteria described above. Two reviewers independently screened the studies in a blinded manner to ensure impartiality and minimize bias (KB and MK). Disagreements were resolved through discussion, and if consensus could not be reached, a third reviewer (KV) was consulted. Common reasons included lack of control group, observational design, and non-English language publications. The quality of the included studies was critically assessed using the Academy of Nutrition and Dietetics Quality Criteria Checklist for Primary Research (25). This instrument was specifically chosen for its detailed criteria tailored to the methodological nuances of dietary intervention studies. It provides a comprehensive evaluation across key bias domains—including selection, performance, detection, attrition, and reporting bias—that is comparable to widely used tools like the Cochrane Risk of Bias (RoB 2) tool. Based on this evaluation, each study was classified as positive (met >80% of quality criteria), neutral (met 50–80%), or negative (met <50%) to allow for balanced comparisons.

### 2.4 Data items

We sought data on key outcomes including disease activity, inflammatory biomarkers, and immune response changes. Disease activity was assessed using standardized scores such as Disease Activity Score-28 (DAS28), Bath Ankylosing Spondylitis Disease Activity Index (BASDAI), Ankylosing Spondylitis Disease Activity Score (ASDAS), Disease Activity in Psoriatic Arthritis Score (DAPSA). Inflammatory biomarkers included CRP, erythrocyte sedimentation rate (ESR), IL-6, TNF-α, and other cytokine levels. Immune response changes were analyzed through markers such as IL-17 expression, FoxP3 gene expression, and alterations in gut microbiota composition.

Other variables collected included participant characteristics (e.g., age, gender, BMI, disease severity, and duration), intervention details (e.g., type, dosage, and duration of supplementation or diet), and trial design features (e.g., randomization, blinding, and control group characteristics). Data on funding sources and potential conflicts of interest were extracted where available.

Missing or unclear data were systematically addressed to minimize bias. If participant-level data were incomplete (e.g., unreported dropouts), the study was classified as having a high risk of attrition bias. Studies lacking essential intervention details (e.g., dosage, administration method) were categorized as unclear and excluded from comparative analyses unless additional information was retrievable from Supplementary material. To mitigate missing data issues, study protocols were cross-referenced, and authors were contacted when feasible. For studies with missing outcome data, a predefined protocol was applied: (1) If dropouts were unreported, the study was categorized as having a high risk of attrition bias; (2) Studies missing essential intervention details were excluded unless further information was available. Missing data were clarified through study protocols or direct author correspondence whenever possible.

### 2.5 Synthesis methods

The eligibility of studies for synthesis was determined based on the intervention characteristics and their relevance to the planned research objectives. Studies were tabulated by intervention type, population, and reported outcomes. These characteristics were compared to predefined inclusion criteria (as outlined in the “Inclusion and Exclusion Criteria” subsection). Missing data were handled by excluding studies with insufficient reporting for synthesis. No data conversions were performed due to the lack of access to raw datasets. Results of individual studies were tabulated in summary tables (Tables 1–3), detailing key characteristics, interventions, outcomes, and main findings. Results were also narratively synthesized and highlighted in the text for clarity. A narrative synthesis was conducted due to significant clinical and methodological heterogeneity observed across studies. A meta-analysis was not performed as this high heterogeneity—present even within seemingly comparable intervention subgroups — precluded meaningful statistical aggregation. Key sources of heterogeneity included wide variations in intervention designs (e.g., diverse diets, supplements, probiotics), variable dosages, differing study durations, and inconsistent comparator groups. Furthermore, the incomplete reporting of data in many primary studies (e.g., lack of mean changes and standard deviations for key outcomes) made it infeasible to calculate the necessary effect sizes for quantitative pooling. Heterogeneity was therefore explored narratively by categorizing studies based on intervention type (e.g., supplements, probiotics, dietary regimens) and outcome measures (e.g., disease activity, inflammatory biomarkers). The risk of bias due to missing results was assessed by examining the completeness of reported data in each study and excluding studies with insufficient information for outcome evaluation. Reporting bias was minimized by strictly adhering to inclusion criteria and conducting independent screening by two reviewers. Certainty in the evidence for each outcome was evaluated using the Academy of Nutrition and Dietetics Quality Criteria Checklist for Primary Research. Studies were classified as positive, neutral, or negative based on adherence to quality standards and transparency in reporting. To enhance transparency, systematic review was registered in the PROSPERO database (registration number: CRD420251010982).

**Table 1:** Key features of the included studies, subdivided according to chronic arthritis subtype—rheumatoid arthritis.

Study	Sample Size (*n*)	Interventions, controls and outcome	Duration	Reported results	Main findings	Quality assessment rating
Aryaeian et al. (26)	70	Intervention: 1,500 mg ginger powder daily Control: placebo daily Outcome: immune response	12 weeks	DAS28 – Ginger group (before/after): 4.73 ± 0.27 vs. 3.44 ± 0.30; *p* = 0.001 – Placebo group (before/after): 4.51 ± 0.27 vs. 4.30 ± 0.33; *p* = 0.18 Between-group comparison: *p* = 0.003=	After the intervention (ginger or placebo), there was a statistically significant drop in DAS28 both within the ginger group and between the two groups (*p =* 0.001 and *p =* 0.003, respectively) Within the ginger group (*p* < 0.05) and between two groups (*p* < 0.05) a substantial increase in FoxP3 gene expression was shown There was a substantial drop in T-bet within the ginger group (*p* < 0.05) and the difference in RORγt gene expression between the two groups was significant (*p =* 0.02), however RORγt gene expression increased non-significantly in the placebo group (*p =* 0.055) whereas it decreased not significantly in the ginger group (*p =* 0.07) PPAR-γ gene expression was considerably upregulated in the ginger group (*p =* 0.047); nonetheless, there was no statistically significant difference between the two groups (*p =* 0.12)	+
Fatel et al. (27)	62	Intervention 1: 3 g of fish oil n-3 fatty acids daily Intervention 2: 3 g of fish oil n-3 fatty acids and 500 mL of reduced-calorie cranberry juice daily Control: typical diet Outcome: disease activity and inflammatory biomarkers	90 days	DAS28-CRP – Control group: pre-intervention: 2.96 [2.55–4.12], post-intervention: 2.77 [2.42–3.52]; *p* = NS – Fish oil group: pre-intervention: 3.65 [2.63–4.42], post-intervention: 2.98 [2.47–3.53]; *p* = 0.045 – Fish oil and cranberry juice group: pre-intervention: 2.57 [2.22–3.21], post-intervention: 1.90 [1.64–2.36]; *p* = 0.001 ESR – Control group: pre-intervention: 28.0 [11.5–39.5], post-intervention: 19.0 [13.0–39.5]: *p* = NS – Fish oil group: pre-intervention: 21.5 [11.5–39.0], post-intervention: 26.5 [21.0–45.0]; *p* = NS – Cranberry juice group: pre-intervention: 16.0 [10.0–27.0], post-intervention: 11.0 [5.5–29.0]; *p* = 0.033 CRP – Control group: pre-intervention: 5.5 [2.0–9.5], post-intervention: 3.9 [1.7–11.1]; *p* = NS – Fish oil group: pre-intervention: 3.7 [1.9–5.5], post-intervention: 7.1 [3.9–12.6]; *p* = NS – Cranberry juice group: pre-intervention: 3.7 [1.7–7.5], post-intervention: 2.5 [1.9–5.1]; *p* = 0.002 IL-6 – Control group: pre-intervention: 6.34 [2.74–18.91], post-intervention: 3.25 [1.27–12.99]; *p* = NS – Fish oil group: pre-intervention: 5.46 [2.59–15.21], post-intervention: 10.18 [4.29–20.62]; *p* = NS Cranberry juice group: pre-intervention: 3.24 [1.00–5.51], post-intervention: 1.00 [1.00–2.14]; *p* = 0.045	The group that was given both cranberry juice and fish oil had lower levels of interleukin-6 (*p =* 0.045), adiponectin (*p =* 0.021), DAS28-CRP (*p =* 0.001), CRP (*p =* 0.002), and ESR (*p =* 0.033) Adiponectin (*p =* 0.023) and DAS28-CRP (*p =* 0.026) were lower in the group that received fish oil alone The group that received both fish oil and cranberry treatment saw decreases (*p* < 0.05) in both CRP and ESR when compared to the control group and the group that received fish oil alone When the fish oil and cranberry group was compared to the control group, a decrease in DAS28-CRP was seen	+
Ghavipour et al. (44)	55	Intervention: 2 capsules of 250 mg pomegranate (POMx) daily Control: 2 capsules of 250 mg cellulose daily Outcome: disease activity and inflammatory biomarkers	8 weeks	DAS28 – POMx group: baseline: 4.9 ± 0.8, change at day 56: −0.9 ± 0.8 – Placebo group: baseline: 4.7 ± 1.1, change at day 56: 0.1 ± 0.5 – Between-group comparison: *p* < 0.001 ESR – POMx group: baseline: 29.0 ± 15.6, сhange at day 56: −4.3 ± 11.0 – Placebo group: baseline: 30.6 ± 19.6, сhange at day 56: 3.5 ± 15.9 Between-group comparison: *p* = 0.03	The DAS28 score was considerably lowered (*p =* 0.001) by the POMx supplement, which may have contributed to a decrease in the number of swollen (*p =* 0.001) and painful joints (*p =* 0.001), pain intensity (*p =* 0.003), and ESR levels (*p =* 0.03) Moreover, POMx consumption raised GPx and reduced morning stiffness (*p =* 0.04) and HAQ score (*p =* 0.007)	+
Gopinath and Danda (45)	121	Intervention: calcium carbonate—1,25 dihydroxy vitamin D combination Control: 1,000 mg of calcium carbonate Outcome: disease activity	3 months	DAS28 – Vitamin D arm: 6.25 ± 1.21– Calcium carbonate arm: 5.91 ± 1.39 ESR – Vitamin D arm: 51.6 ± 27.5– Calcium carbonate arm: 53.92 ± 30.5 CRP – Vitamin D arm: 16.1 ± 21.49– Calcium carbonate arm: 23.6 ± 34.8 – The article does not provide data for post-intervention ESR, CRP, or DAS28 Pan Relief – Reduction in VAS score at the end of 3 months (%):– Vitamin D arm: 50 (0–100) Calcium carbonate arm: 30 (0–100). *p*-value: reduction in VAS score at the end of 3 months: *p* = 0.006	Higher pain alleviation was reported by patients on the vitamin D group (*n* = 59) compared to the control group (*n* = 62) (50% vs. 30%, *p =* 0.006)	+
Esalatmanesh et al. (47)	74	Intervention: 600 mg of N-acetylcysteine (NAC) twice daily Control: placebo twice daily Outcome: disease activity, blood lipids/sugar, inflammatory biomarkers	3 months	DAS28 – NAC group: baseline: 5.96 ± 1.05, after 12 weeks: 2.92 ± 1.48 – Placebo group: baseline: 6.28 ± 1.27, after 12 weeks: 3.36 ± 1.66 – *p*-value (within group): *p* < 0.001 – Between-group comparison: *p* = 0.295 ESR – NAC group: baseline: 30.59 ± 25.33, after 12 weeks: 16.15 ± 11.82 – Placebo group: baseline: 36.75 ± 26.75, after 12 weeks: 22.97 ± 19.65 – *p*-value (within group): NAC group = 0.004, placebo group: *p* < 0.001 – Between-group comparison: *p* = 0.142 hs-CRP – NAC group: baseline: 14.0 [2.0–30.0], after 12 weeks: 4.50 [2.0–12.25] – Placebo group: baseline: 9.50 [2.0–22.75], after 12 weeks: 3.0 [2.0–12.75] *p*-value (within group): NAC group: *p* = 0.006, Placebo group: *p* = 0.122. Between-group comparison: *p* = 0.353	NAC significantly increased GPx activity (*p =* 0.015) and high-density lipoprotein cholesterol (HDL-C) level (*p =* 0.001) and significantly decreased morning stiffness (*p* < 0.001), DAS-28 (*p* < 0.001), ESR (*p =* 0.004), MDA (*p* < 0.001), NO (*p* < 0.001), hs-CRP (*p =* 0.006), FBS (*p* < 0.001), and low-density lipoprotein cholesterol (LDL-C) (*p =* 0.023) Following the treatment, significant variations were observed solely in serum NO (*p =* 0.003), FBS (*p =* 0.010), and HDL-C (*p* < 0.001), after adjusting for baseline measurements between the two groups While significant reductions were seen within both groups for DAS-28, morning stiffness, and ESR, there were no significant differences between the two groups for these outcomes when adjusting for baseline measures	+
Bae et al. (48)	32	Group 1: quercetin + vitamin C (166 mg/133 mg/capsule) 3 capsules/day Group 2: lipoic acid (300 mg/capsule), 3 capsules/day Group 3: placebo 3 capsules/day Outcome: disease activity and inflammatory biomarkers	16 weeks	CRP – Placebo group: baseline: 0.85 [0.28–4.00], after 4 weeks: 2.01 [0.16–4.81], change: 0.060 [−3.95–0.90] – Quercetin group: baseline: 1.05 [0.22–6.44], after 4 weeks: 1.10 [0.29–3.51], change: +0.045 – Lipoic acid group: baseline: 0.84 [0.14–4.28], after 4 weeks: 0.54 [0.22–3.23], change: 0.08 [−0.04–0.27] – Comparison between treatment change from baseline: *p* = 0.24 IL-1β – Placebo group: baseline: 2.41 ± 0.67, after 4 weeks: 2.35 ± 0.57 pg/mL, change: 0.04 ± 0.55 – Quercetin group: baseline: 2.44 ± 0.17, after 4 weeks: 2.40 ± 0.16, change: −0.04 ± 0.14 – Lipoic acid group: baseline: 2.47 ± 0.24, after 4 weeks: 2.41 ± 0.19, change: −0.05 ± 0.28 – Comparison between treatment change from baseline: *p* = 0.65 TNF-α – Placebo group: baseline: 3.50 ± 1.96, after 4 weeks: 3.44 ± 1.75 pg/mL, change: −0.06 ± 0.28 – Quercetin group: baseline: 3.36 ± 1.35, after 4 weeks: 3.32 ± 1.32, change: −0.04 ± 0.23 – Lipoic acid group: baseline: 3.37 ± 1.46, after 4 weeks: 3.33 ± 1.41, change: −0.04 ± 0.16 – Comparison between treatment change from baseline: *p* = 0.94 IL-6 – Placebo group: baseline: 3.91 ± 1.53, after 4 weeks: 3.94 ± 1.73, change: +0.03 ± 0.95 – Quercetin group: baseline: 4.34 ± 2.00, after 4 weeks: 3.79 ± 1.44, change: −0.54 ± 1.10 – Lipoic acid group: baseline: 4.24 ± 2.05, after 4 weeks: 4.22 ± 1.82 pg/mL, change: −0.02 ± 1.40 Comparison between treatment change from baseline (week 0): *p* = 0.38	Before the patient began the subsequent supplementing, there was a 2-week wash-out interval following each 4-week treatment cycle There were no discernible variations in the blood levels of pro-inflammatory cytokines and CRP among the various therapies Despite quercetin supplementation’s tendency to lower VAS, the scores of illness severity assessments did not differ substantially between treatments	∅
Bahadori et al. (28)	23	Intervention: 0.2 g of fish oil emulsion/kg IV followed by 0.05 g of fish oil/kg orally Control: 0.9% saline (placebo) infusion IV followed by paraffin wax (placebo) ingested orally Outcome: disease activity	22 weeks	SJC – Baseline: ω-3 FA: 10 [6–14], placebo: 14 [6–37] – Week 1: ω-3 FA: 3 [0–7], placebo: 8 [1–19]; *p* = 0.002 – Week 2: ω-3 FA: 1 [0–9], placebo: 7 [0–16]; *p* = 0.046 – Week 4: ω-3 FA: 2 [0–12], placebo: 10 [2–27]; *p* = 0.012 – Week 11: ω-3 FA: 1.5 [0–13], placebo: 9 [1–28]; *p* = 0.15 – Week 22: ω-3 FA: 1 [0–5], placebo: 8 [4–10]; *p* = 0.006 TJC – Baseline: ω-3 FA: 18 [10–37], placebo: 17 [8–41] – Week 1: ω-3 FA: 8 [1–27], placebo: 12.5 [2–19]; *p* = 0.65 – Week 2: ω-3 FA: 1 [0–31], placebo: 7 [5–25]; *p* = 0.12 – Week 4: ω-3 FA: 5 [0–31], placebo: 15 [10–36]; *p* = 0.007 – Week 11: ω-3 FA: 3.5 [0–20], placebo: 14 [3–26]; *p* = 0.11 – Week 22: ω-3 FA: 2.5 [0–12], placebo: 8 [5–11]; *p* = 0.033 ESR and CRP – CRP levels at week 1: ω-3 FA group had 2.2 ± 1.2 g/L, placebo group had 4.4 ± 4.3. Between-group comparison: *p* = 0.12 – ESR levels at baseline: ω-3 FA group 5.1 ± 3.0, placebo group 5.5 ± 3.6. Between-group comparison: *p* = 0.80 The study states that CRP and ESR levels were not significantly different between the groups throughout the study, and no further numerical data for later weeks are provided in the article for these markers	The ω-3 FA group had a substantially lower swollen joint count after 1 week of infusion (*p =* 0.002) and 2 weeks of infusion (*p =* 0.046) when compared to the placebo group Additionally, the ω-3 FA group tended to have a reduced tender joint count, however this difference was not statistically significant During and after oral treatment, the ω-3 FA group experienced considerably less swollen and sore joint counts than the placebo group	∅
Berbert et al. (29)	55	Intervention 1: fish oil ω-3 fatty acids (3 g/day) (G2) Intervention 2: fish oil ω-3 fatty acids (3 g/day) and 9.6 mL of olive oil (G3) Control: placebo (soy oil) (G1) Outcome: disease activity	24 weeks	Ritchie’s articular index – Baseline: G1: 6.9 ± 5.4, G2: 15.8 ± 9.9, G3: 15.9 ± 12.6 – After 12 weeks: G1: 5.5 ± 7.5, G2: 7.6 ± 6.7, G3: 5.8 ± 8.2 – After 24 weeks: G1: 5.2 ± 4.4, G2: 3.6 ± 2.4, G3: 1.2 ± 2.3 – Between-group comparison, 24 weeks: *p* < 0.05 (for G2 and G3 vs. G1) RF – Baseline: G1: 206 ± 189, G2: 243 ± 343, G3: 302 ± 321 – After 12 weeks: G1: 176 ± 166, G2: 246 ± 352, G3: 268 ± 329 – After 24 weeks: G1: 205 ± 178, G2: 201 ± 295, G3: 208 ± 298 – Between-group comparison: *p* < 0.05 (for G3 vs. G1 at 24 weeks) ESR – Baseline: G1: 29.1 ± 18.1, G2: 21.6 ± 20.0, G3: 29.4 ± 23.2 – After 12 weeks: G1: 30.2 ± 16.9, G2: 22.9 ± 18.9, G3: 32.2 ± 23.5 – After 24 weeks: G1: 35.7 ± 21.8, G2: 25.5 ± 16.1, G3: 26.8 ± 20.0 – Between-group comparison, 24 weeks: *p* > 0.05 CRP – Baseline: G1: 15.5 ± 21.1, G2: 17.9 ± 20.3, G3: 24.8 ± 31.6 – After 12 weeks: G1: 20.5 ± 14.7, G2: 15.5 ± 11.5, G3: 21.4 ± 25.2 – After 24 weeks: G1: 18.1 ± 15.8, G2: 19.5 ± 22.3, G3: 17.4 ± 23.8 Between-group comparison, 24 weeks: *p* > 0.05	Eating fish oil high in n-3 PUFA fatty acids improved a number of clinical indicators (joint pain intensity, right and left handgrip strength, duration of morning stiffness, onset of fatigue) (*p* < 0.05) When olive oil and fish oil supplements were combined, patients’ improvements were more pronounced and premature	+
Dawczynski et al. (30)	38	Intervention: microalgae oil from *Schizochytrium* sp. (2.1 g DHA/day) Control: sunflower oil (placebo) Outcome: disease activity	30 weeks	DAS28 – Verum group: week 0: 4.25 ± 0.97, week 10: 3.88 ± 1.17, change from baseline: −0.36 ± 0.96 – Placebo group: week 0: 3.99 ± 0.91, week 10: 4.13 ± 1.2, change from baseline: 0.14 ± 0.9 – Between-group comparison: *p* = 0.085 ESR – Verum group: week 0: 24.80 ± 19.02, week 10: 26.92 ± 21.69, change from baseline: 2.12 ± 8.00 – Placebo group: week 0: 23.50 ± 14.45, week 10: 25.79 ± 20.68, change from baseline: 2.29 ± 14.07 – Between-group comparison: *p* = 0.925 CRP – Verum group: week 0: 9.03 ± 9.84, week 10: 7.57 ± 7.62, change from baseline: −1.47 ± 7.16 – Placebo group: weeks 0: 6.20 ± 3.90, weeks 10: 6.51 ± 5.58, change from baseline: 0.31 ± 5.24 Between-group comparison: *p* = 0.829	The amount of sensitive and swollen joints (68/66) decreased from 13.9 ± 7.4 to 9.9 ± 7.0 (*p* < 0.010), the total DAS28 decreased from 4.3 ± 1.0 to 3.9 ± 1.2 (*p* < 0.072), and the ultrasound score (US-7) decreased from 15.1 ± 9.5 to 12.4 ± 7.0 (*p* < 0.160) in response to daily DHA ingestion Daily consumption of DHA from microalgae oil resulted in a significant reduction in the sum of tender and swollen joints, and the number of tender joints (DAS28), compared to placebo. DHA supplementation also caused a shift in the balance of lipid mediators towards an anti-inflammatory state	+
Dawczynski et al. (31)	45	Intervention: n-3 LC-PUFA-supplemented dairy Control: placebo Outcome: inflammatory biomarkers, blood lipids	8 months	DAS28 – Intervention group: baseline: 4.45 ± 1.05, after 12 weeks: 4.32 ± 1.11 – Control group: baseline: 4.18 ± 1.11, after 12 weeks: 4.24 ± 0.80 – Between-group comparison: *p* = NS ESR – Intervention group: baseline: 22.4 ± 20.4, after 12 weeks: 23.8 ± 20.0 – Control group: baseline: 17.5 ± 11.1, after 12 weeks: 19.4 ± 11.4 – Between-group comparison: *p* = NS CRP – Intervention group: baseline: 12.2 ± 10.5, after 12 weeks: 14.4 ± 14.0 – Control group: baseline: 9.81 ± 8.86, after 12 weeks: 7.74 ± 4.13 Between-group comparison: *p* ≤ 0.05	A long-term intervention with n-3 long-chain PUFA-supplemented dairy products improved serum lipids by increasing HDL and lowering lipoprotein a The study provided evidence of cardioprotective effects, but no significant improvement in overall disease activity markers such as DAS28, tender joints, swollen joints, CRP, or ESR was observed	+
Geusens et al. (32)	90	Intervention 1: 2.6 g ω-3 fatty acid daily Intervention 2: 1.3 g ω-3 fatty acids + 3 g olive oil daily Control: 6 g olive oil daily Outcome: disease activity	12 months	Changes at 12 months Ritchie articular index – Placebo: −15 ± 4 – 1.3 g/day ω3: −9 ± 3 – 2.6 g/day ω3: −14 ± 4 RF – Placebo: +24 ± 8 – 1.3 g/day ω3: +51 ± 18 – 2.6 g/day ω3: +11 ± 7 Baseline ESR – Placebo: 23 ± 3 – 1.3 g/day ω-3: 22 ± 3 – 2.6 g/day ω-3: 33 ± 6 – Changes after intervention: the study noted that ESR tended to increase in the placebo group, was not significantly altered in the 1.3 g/day ω-3 group and tended to decrease in the 2.6 g/day ω-3 group. *p*-value (between groups): NS Specific post-intervention values are not provided in the article’s tables.	Significant improvement in pain assessment and overall patient evaluation in those receiving 2.6 g ω-3 fatty acid daily (*p* < 0.05) Enhancement and decrease of concurrent anti-rheumatic drugs were considerably higher when taking 2.6 g of n-3 PUFA fatty acid daily	∅
Hosseini et al. (33)	42	Intervention: fish oil supplementation Control: no fish oil Outcome: disease activity and inflammatory biomarkers	8 weeks	ESR – Before supplementation: 40.45 ± 3.93 – After 4 weeks: 23.07 ± 4.31; *p* = NS – After 8 weeks: 24.77 ± 3.89; *p* = 0.003 CRP – Before supplementation: 15.34 ± 4.04 – After 4 weeks:11.67 ± 3.62; *p* = NS – After 8 weeks: 8.25 ± 3.17; *p* = 0.002 RF – Before supplementation: 15.97 ± 3.42 – After 4 weeks: 13.22 ± 3.34; *p* = 0.004 – After 8 weeks: 12.08 ± 3.60; *p* = 0.009 Clinical findings (percentage of patients showing improvement): – Number of inflamed joints: 64.2% after 4 weeks and 80.9% after 8 weeks. Patient global assessment: 80.9% after 4 weeks and 95.2% after 8 weeks.	RF (*p =* 0.009), ESR (*p =* 0.003), and serum CRP (*p =* 0.002) all exhibited substantial improvement after 8 weeks, while only RF (*p =* 0.004) showed significant improvement after 4 weeks.	+
Kremer et al. (34)	49	Intervention: 2 different doses of fish oil Control: olive oil Outcome: disease activity and immune response	24 weeks	TJC Mean change from baseline and 95% confidence interval: – Olive oil: baseline 5.8 (2.0–9.0), 6 weeks: −0.7 (−2.6, 1.3), 12 weeks: −0.4 (−1.9, 2.8), 18 weeks: −0.8 (−2.4, 0.8), 24 weeks: 0.4 (−2.3, 3.2) 36 weeks: −0.3 (−2.8, 2.2) – Low-dose fish oil (*n* = 20): baseline 6.0 (3.3–8.7), 6 weeks: 0.1 (−2.1, 2.2), 12 weeks: −1.7 (−3.9, 0.5), 18 weeks: −1.1 (−2.8, 0.7), 24 weeks: −1.9 (−3.7, 0.0) 36 weeks: −1.0 (−3.2, 1.2) – High-dose fish oil: baseline 5.4 (1.8–8.9), 6 weeks: −1.1 (−3.3, 1.2), 12 weeks: −2.4 (−4.8, 0.1), 18 weeks: −2.6 (−5.1, 0.0), 24 weeks: −1.7 (−3.1, 0.2), 36 weeks: −1.8 (−4.0, 0.3) JSC Mean change from baseline and 95% confidence interval: – Olive oil: baseline 16.3 (10.6–22.2), 6 weeks: −0.8 (−3.5, 1.9), 12 weeks: −2.8 (−6.3, 0.6), 18 weeks: −2.6 (−6.5, 1.3), 24 weeks: −2.4 (−5.8, 0.9), 36 weeks: −2.4 (−6.3, 1.6) – Low-dose fish oil: baseline 14.4 (11.3–17.5), 6 weeks: −0.8 (−2.5, 0.8), 12 weeks: −2.7 (−4.4, −1.0), 18 weeks: −3.6 (−5.6, −1.5), 24 weeks: −4.1 (−6.9, 1.8), 36 weeks: −3.6 (−6.1, 1.1) – High-dose fish oil: baseline 13.0 (10.7–15.3), 6 weeks: −0.4 (−3.4, 1.7), 12 weeks: −2.9 (−4.0, −1.8), 18 weeks: −2.3 (−3.9, −0.7), 24 weeks: −2.8 (−5.0, −0.7), 36 weeks: −1.4 (−3.5, 0.8) IL-1 Mean change from baseline at week 24: – Olive oil: −243.1 (−540.4, 54.2) – Low-dose fish oil: −239.9 (−490.4, 10.6) – High-dose fish oil: −416.2 (−623.0, 209.4) IL-2 Mean change from baseline at week 24: – Olive oil: 34 (−7.6, 92.9) – Low-dose fish oil: 25 (−18.7, 68.7) – High-dose fish oil: 1.5 (−21.3, 24.2) LTB4 Mean change from baseline at week 24: – Olive oil: −1.81 (−4.43, −0.81) – Low-dose fish oil: −3.88 (−5.73, −2.03) – High-dose fish oil: −4.13 (−7.73, −0.53) No significant changes were observed in ESR or rheumatoid factor titer in any group	The clinical benefits of n-3 PUFA fatty acid dietary supplements were more commonly observed in patients consuming higher dosages of fish oil. Significant improvements in tender and swollen joints, as well as a decrease in the production of pro-inflammatory markers, were observed in the fish oil groups but not in the olive oil group The study found that the mean change from baseline was significant for most clinical parameters in the high-dose fish oil group, and for some in the low-dose group, while the olive oil group showed little to no significant change	+
Kremer et al. (35)	66	Intervention: fish oil supplementation Control: placebo diclofenac Outcome: NSAID continuation, disease activity, immune response	48 weeks	TJC Change to maximum duration of fish oil (8 weeks after stopping diclofenac): – Fish oil group: −7.8 ± 2.6; comparison with baseline: *p* = 0.01 – Corn oil group: −6.4 ± 2.2; comparison with baseline: *p* = 0.78 – Between-group comparison: *p* = 0.043 SJC Change to maximum duration of fish oil: – Fish oil group: −4.7 ± 2.7; comparison with baseline: *p* = 0.10 – Corn oil group: −5.6 ± 1.7; comparison with baseline: *p* = 0.004 IL-1β Change to maximum duration of diclofenac: – Fish oil group: −7.7 ± 3.1; comparison with baseline: *p* = 0.026 – Corn oil group: no significant change from baseline and with fish oil From the baseline evaluation to the maximum duration of fish oil at week 26 or 30, there was a significant increase in TNFα levels in the patients taking fish oil (45.1 ± 13.6; *p* = 0.013) and in those taking corn oil (65.8 ± 27.5; *p* = 0.038). No significant changes in cytokines were observed when patients taking fish oil were compared with those taking corn oil at this time	Compared to baseline, fish oil improves clinical markers of disease activity, such as the number of painful joints There is a correlation with notable drops in IL-1B levels from baseline Some fish oil users are able to stop taking NSAIDs without experiencing a flare-up of their illness	∅
Lorenzetti et al. (49)	40	Intervention: LD-1227 Control: n-3 PUFA Outcome: disease activity, inflammatory biomarkers	12 weeks	ACR response rates – ACR20 response: 81.0% in LD-1227 group vs. 44% in n-3 PUFA group – ACR50 response: 62% in LD-1227 group vs. 31% in n-3 PUFA group – *p*-value (for ACR responses): *p* < 0.01 for both ACR20 and ACR50 responses DAS28 – LD-1227: baseline: 4.3 ± 0.9, post-intervention: 2.2 ± 1.2 – n-3 PUFA: baseline: 4.0 ± 1.4, post-intervention: 3.5 ± 0.9 – Between-group comparison: *p* < 0.05 CRP – LD-1227: baseline: 10.2 ± 6.4, post-intervention: 5.01 ± 1.17 – n-3 PUFA: baseline: 9.1 ± 4.3, post-intervention: 8.29 ± 1.31 Between-group comparison: *p* < 0.01	At 12 weeks, 81.0% of the LD-1227 group and 44% of the n-3 PUFA group had a favorable response to ACR20 (*p* < 0.01) The VAS scale, HAQ score, morning stiffness, and tender spots all significantly improved in the LD-1227-treated group (*p* < 0.01 vs. control and *p* < 0.05 versus n-3 PUFA) CRP, IL-6, TNF-α, IL-1β, CXCL1, IFNγ, IL-15, and IP-10 levels are lower in the LD-1227 group, and there is a notable downregulation of associated gene expressions	∅
Park et al. (36)	109	Intervention: n-3 PUFA (2.090 g of EPA and 1.165 g of DHA) Control: high-oleic-acid sunflower oil Outcome: disease activity, NSAID continuation	16 weeks	NSAIDs (subgroup >55 kg): – Mean difference (change from baseline): approximately −12 mg; *p* = 0.043 LTB4 (subgroup >55 kg): – Mean difference (change from baseline): approximately −0.3 nmol/L; *p* = 0.021 TNF-α – n-3 PUFA: baseline: 210.55 ± 457.85, 16 weeks: 202.68 ± 448.37 – Placebo: baseline: 138.65 ± 301.54, 16 weeks: 124.40 ± 310.70 – Between-group comparison: *p* = 0.802 IL-6 – n-3 PUFA: baseline: 455.26 ± 518.46, 16 weeks: 506.46 ± 542.81 – Placebo: baseline: 320.63 ± 377.49, 16 weeks: 412.31 ± 394.65 – Between-group comparison: *p* = 0.697 hs-CRP – n-3 PUFA: baseline: 25.97 ± 32.17, 16 weeks: 31.22 ± 42.90 – Placebo: baseline: 32.19 ± 51.44, 16 weeks: 43.95 ± 89.33 Between-group comparison: *p* = 0.690	Supplementing with n-3 PUFAs had no significant effect on nonsteroidal anti-inflammatory drug requirements, clinical symptoms of RA, or the concentration of inflammatory markers However, in a subgroup analysis of patients weighing more than 55 kg, n-3 PUFA significantly decreased NSAID requirements and leukotriene B4 levels	∅
Proudman et al. (37)	139	Intervention: high dose fish oil Control: low dose fish oil Outcome: disease activity	1 year	ACR remission: – EPA: HR: 1.12, 95% CI: 1.02, 1.23; *p* = 0.02 – EPA (corrected): HR: 1.12, 95% CI: 1.02, 1.24; *p* = 0.02 – DHA: HR: 1.12, 95% CI: 0.95, 1.32; *p* = 0.17 – DHA (corrected): HR: 1.10, 95% CI: 0.93, 1.31; *p* = 0.27 – EPA + DHA: HR: 1.07, 95% CI: 1.01, 1.14; *p* = 0.04 – EPA + DHA (corrected): HR: 1.07, 95% CI: 1.00, 1.14; *p* = 0.05 DAS28 remission: EPA: HR: 1.03, 95% CI: 0.95, 1.12; *p* = 0.47 – EPA (corrected): HR: 1.03, 95% CI: 0.95, 1.12; *p* = 0.47 – DHA: HR: 1.03, 95% CI: 0.91, 1.17; *p* = 0.65 – DHA (corrected): HR: 1.03, 95% CI: 0.90, 1.18; *p* = 0.65 – EPA + DHA: HR: 1.02, 95% CI: 0.97, 1.07; *p* = 0.51 – EPA + DHA (corrected): HR: 1.02, 95% CI: 0.97, 1.07; *p* = 0.51	A one-unit rise in EPA (1% total fatty acids) was linked to a 12% increase in the probability of remission at any point throughout the study period (hazard ratio (HR) = 1·12; 95% confidence interval (CI): 1·02, 1·23; *p* = 0·02). This suggests that plasma PL EPA was connected to the time to remission	+
Remans et al. (38)	66	Intervention: EPA, DHA, GLA supplement Control: placebo Outcome: disease activity	4 months	DAS28 – Placebo: baseline: 5.14 ± 1.05, change at 2 months: 0.22 ± 0.74, change at 4 months: 0.21 ± 0.93 – Supplement: baseline: 5.36 ± 0.92, change at 2 months: −0.01 ± 0.82, change at 4 months: 0.22 ± 0.77 ESR – Placebo: baseline: 29 ± 23, change at 2 months: 4 ± 12, change at 4 months: 2 ± 9 – Supplement: baseline: 30 ± 21, change at 2 months: 1 ± 10, change at 4 months: 4 ± 10 CRP – Placebo: baseline: 18.6 ± 19.8, change at 2 months: 0.0 ± 10.5, change at 4 months: −0.4 ± 11.2 Supplement: baseline: 14.8 ± 12.4, change at 2 months: 1.0 ± 10.6, change at 4 months: 2.6 ± 10.0	Neither group’s painful joint count nor any other clinical indicator showed a discernible difference from baseline Patients who were supplemented with nutrients showed substantial increases in plasma concentrations of vitamin E (*p =* 0.015), as well as EPA, DHA, and docosapentaenoic acid, along with concurrent decreases in arachidonic acid (*p =* 0.01), but not those who received a placebo	+
Soubrier et al. (46)	59	Intervention: vitamin D ampules Control: placebo Outcome: disease activity, inflammatory biomarkers	6 months	HAQ score Mean change: Overall, HAQ baseline 1.05 ± 0.74 – Placebo group: +0.08 ± 0.25 – VitD group: −0.03 ± 0.23 Comparison between groups, unadjusted: *p* = 0.11 Comparison between groups, adjusted: *p* = 0.046 ESR (mean change): the article does not provide the exact numerical difference, but it is noted that after adjusting for relevant parameters, a significant improvement was achieved in the vitamin D group compared to placebo Comparison between groups, adjusted: *p* = 0.002 CRP (mean change): similarly, the exact numerical difference is not provided, but a significant improvement was achieved in the vitamin D group compared to placebo Comparison between groups: *p* = 0.04 DAS-ESR baseline 3.7 ± 0.8 DAS-CRP baseline 3.5 ± 0.8 The numerical data, the article does not provide the specific figures for the mean or median change in DAS28 scores. It only discusses the results qualitatively. After adjusting for age, gender, season, and initial vitamin D status, no significant difference was found in DAS28 between the groups. A trend toward a superior improvement in DAS28-ESR was observed in the vitamin D group, but this difference did not reach statistical significance	At 6 months, HAQ score for the placebo group increased by an average of 0.08 (±0.25), while the vitamin D group’s score slightly decreased by an average of 0.03 (±0.23). This initial difference was not statistically significant (*p =* 0.11) after adjusting for factors such as age, gender, season, and initial vitamin D status, the difference between the groups became statistically significant (*p =* 0.046) ESR and CRP (*p =* 0.002 and *p =* 0.04, respectively) were the only secondary criteria that showed a significant difference between the 2 groups after accounting for age, gender, season, and initial vitD status	+
van der Tempel et al. (40)	16	Intervention: fish oil Control: coconut oil (placebo) Outcome: inflammatory biomarkers and disease activity	24 weeks	Joint pain index (points): – None (pre-diet): 33 ± 7 – Fish oil: 29 ± 7 – Coconut oil: 42 ± 9; *p* > 0.05 Joint swelling index (points): – None (pre-diet): 10 ± 2 – Fish oil: 2 ± 1 – Coconut oil: 8 ± 3; *p* = 0.01 ESR – None (pre-diet): 30 [19–98] – Fish oil: 34 [14–80] – Coconut oil: 40 [11–70]; *p* > 0.05 CRP – None (pre-diet): 30 [3–121] – Fish oil: 17 [3–69] – Coconut oil: 21 [5–71]; *p* > 0.05 Leucotriene B4: – None (pre-diet): 149 ± 13 – Fish oil: 123 ± 10 – Coconut oil: 141 ± 12; *p* < 0.05 Leucotriene B5: – None (pre-diet): 0 ± 0 – Fish oil: 13 ± 2 Coconut oil: 0 ± 0; *p* < 0.01	Preference for fish oil: indicator of joint swelling and length of stiffness in the morning Fish oil intake resulted in a significant increase in eicosapentaenoic acid and docosahexaenoic acid (n-3 PUFA fatty acids) in both plasma cholesterol ester and neutrophil membrane phospholipid fractions (*p* < 0.01), mainly at the cost of omega-6 fatty acids	+
Nordström et al. (43)	22	Intervention: alpha linolenic acid Control: linolenic acid (placebo) Outcome: disease activity and inflammatory biomarkers	3 months	Joint score index – Treatment group: before: 9.3 ± 7.9; after: 9.1 ± 7.5 – Placebo group: before: 11.5 ± 4.2; after: 9.5 ± 4.3 ESR – Treatment group: before: 30.9 ± 24.1, after: 35.7 ± 27.1 – Placebo group: before: 34.2 ± 20.4, after: 32.5 ± 20.5 CRP – Treatment group: before: 17.2 ± 9.6, after: 20.3 ± 12.4 Placebo group: before: 21.7 ± 11.9, after: 21.8 ± 16.8	A 3-month supplementation with alpha-linolenic acid (alpha-LNA) did not result in any significant improvements in clinical or laboratory parameters of rheumatoid arthritis Although serum alpha-LNA levels significantly increased in the treatment group, the concentrations of arachidonic acid (AA), eicosapentaenoic acid (EPA), and docosahexaenoic acid (DHA) did not change	∅
Zamani et al. (57)	54	Intervention: synbiotic capsule Control: placebo Outcome: disease activity and inflammatory biomarkers	8 weeks	DAS28 – Synbiotic group: DAS28 score decreased from a baseline of 4.2 ± 0.7 to 2.6 ± 0.7 at the end of the trial, representing a mean change of −1.6 ± 0.8 – Placebo group: DAS28 score changed from 3.5 ± 0.8 to 3.2 ± 1.1, a mean change of −0.3 ± 0.5 – Comparison between groups: *p* < 0.001 hs-CRP – Synbiotic group: hs-CRP level decreased from a baseline of 6037.0 ± 4839.7 to 4609.2 ± 2711.7, resulting in a mean change of −1427.8 ± 3267.2 – Placebo Group: hs-CRP level increased from 5640.7 ± 5141.0 to 8474.1 ± 6829.7, a mean change of +2833.4 ± 5639.7 Comparison between groups: *p* = 0.001	For 8 weeks, RA patients who took supplements of synbiotics showed improvements in their levels of hs-CRP (*p =* 0.001), DAS-28 (*p* < 0.001), VAS (*p* < 0.001), NO (*p =* 0.008), insulin (*p =* 0.01), HOMA-IR (*p =* 0.03), HOMA-B (*p =* 0.01), and GSH (*p =* 0.005)	+
Alavi et al. (50)	69	Intervention: prebiotic dPP supplement active compound (AC) Control: placebo Outcome: serum glycosylation and disease activity	6 months	DAS28 Difference in means between groups, adjusted for baseline: – AC-Placebo: 0.63 ± 0.23, 95%CI 0.17–1.10; *p* = 0.009 (in favor of placebo) ESR: ratio (AC/placebo): 1.20. 95%CI 0.97–1.47; *p* = 0.09 CRP: ratio (AC/placebo): 1.54, 95%CI 1.05–2.27; *p* = 0.03	The levels of agalactosylated (G0F) glycans were decreased by 12% after supplementation [from 8.10 (0.89) to 7.16 (0.60); *p =* 0.03] There is no noteworthy overall impact on patient outcomes	+
Cannarella et al. (51)	47	Intervention: daily ingestion of probiotics Control: daily ingestion of maltodextrin (placebo) Outcome: disease activity and inflammatory biomarkers	60 days	DAS28 – Placebo: baseline: 3.83 [2.75–4.69], after 60 days: 3.88 [3.29–4.45] – Probiotic: baseline: 3.20 [2.47–4.21], after 60 days: 3.18 [2.49–3.96] – Comparison between groups: *p* > 0.05 hsCRP – Placebo: baseline: 4.00 [2.30–7.40], after 60 days: 2.90 [1.80–14.40]; *p* = 0.626 – Probiotic: baseline: 4.70 [1.50–11.90], after 60 days: 4.60 [2.40–9.30]; *p* = 0.765 – Intergroup change: *p* > 0.05 ESR – Placebo: baseline: 23.00 [9.00–48.50], after 60 days: 29 [12.00–39.00]; *p* = 0.717 – Probiotic: baseline:19.50 [14.50–33.00], after 60 days: 25.00 [16.00–42.00], *p* = 0.197 – Intergroup change: *p* > 0.05 TNF-α: – Change after 60 days: significant reduction in the probiotic group (*p* = 0.004) and in the placebo group (*p* = 0.032) vs. baseline. Intergroup change: a significant difference was noted between the treatments (*p* < 0.05) IL-6: Change after 60 days: significant reduction in the probiotic group (*p* = 0.039 vs. placebo). Intergroup change: *p* = 0.039	The probiotic group exhibited a statistically significant decrease in interleukin 6 plasma levels (*p =* 0.039), tumor necrosis factor-a (*p =* 0.004), and white blood cell count (*p =* 0.012) Interleukin-10, adiponectin, CRP, ESR, ferritin, and DAS28 did not differ from one another When compared to the placebo group, the probiotic group had reduced nitric oxide metabolites (*p =* 0.004), higher sulfhydryl group (*p =* 0.028), and higher overall radical-trapping antioxidant characteristics (*p =* 0.019) There was no difference in lipid hydroperoxide and protein carbonyl across the groups (*p* > 0.05)	∅
Esmaeili et al. (58)	186	Intervention: daily oral synbiotic supplement (1,000 mg) Control: placebo Outcome: disease activity and inflammatory biomarkers	3 months	DAS28 – A significant decrease was observed in both the synbiotic and placebo groups after 3 months (*p* < 0.05). The mean values decreased from approximately 5.8 (synbiotic-0) to 4.8 (synbiotic-3) and rom approximately 5.2 (placebo-0) to 4.5 (placebo-3). There was no overall significant difference between the synbiotic and placebo groups, except for a specific subgroup. The specific subgroup that showed a significant difference was patients receiving 7.5–10 mg MTX and 5–10 mg Pred (*p* < 0.05) ESR – The study found no significant change in ESR in either the synbiotic or placebo groups after 3 months CRP A significant decrease in the CRP level (*p* < 0.05) was noted only in a specific subgroup of the synbiotic group (patients receiving 15–20 mg MTX and 5–10 mg Pred)	After the intervention DAS28 count decreased significantly (*p* < 0.05), but there was no significant change in ESR. A decreased CRP (*p* < 0.05), decreased VAS score (*p* < 0.05) and a decrease of tender joint count and swollen joint count (*p* < 0.05) after intervention was noticed	∅
Hatakka et al. (52)	21	Intervention: 2 capsules of LGG twice daily Control: placebo twice daily Outcome: disease activity	12 months	No. of tender joints – LGG: baseline: 3.7 ± 2.5, treatment period: 2.5 ± 1.7 – Placebo: baseline: 3.0 ± 3.3, treatment period: 2.6 ± 2.4 – LGG vs. placebo: mean difference: −0.3, 95% CI: (−2.2 to 1.7); *p* = 0.784 No. of swollen joints – LGG: baseline: 4.5 ± 5.5, treatment period: 2.1 ± 1.7 – Placebo: baseline: 2.5 ± 3.0, treatment period: 2.2 ± 3.1 – LGG vs. placebo: mean difference: −1.1, 95% CI: (−3.0 to 0.9); *p* = 0.265 ESR – LGG: baseline: 17.3 ± 14.7, treatment period: 20.7 ± 17.3 – Placebo: baseline: 18.2 ± 15.9, treatment period: 17.9 ± 14.4 – LGG vs. placebo: mean difference: 3.6, 95% CI: (−0.7 to 7.9); *p* = 0.095 CRP – LGG: baseline: 1.6 ± 4.6, treatment period: 2.6 ± 3.3 – Placebo: baseline: 5.1 ± 5.7, treatment period: 7.4 ± 8.7 – LGG vs. placebo: mean difference: −1.3, 95% CI: (−6.2 to 3.6); *p* = 0.582 IL-6 – LGG: baseline: 4.6 [2.1–21.3], treatment period: 4.8 [2.2–14.7] – Placebo: baseline: 6.5 [1.7–17.5], treatment period: 6.7 [1.6–35.3] – Comparison between groups: *p* = 0.529 TNF-α – LGG: baseline: 1.9 [0.4–10.3], treatment period: 2.6 [0.5–10.8] – Placebo: baseline: 1.5 [0.4–6.3], treatment period: 1.7 [0.4–5.8] Comparison between groups: *p* = 0.529	Throughout the intervention period, no statistically significant variations were seen in the clinical parameters, biochemical variables, or HAQ index among the study groups	+
Mandel et al. (53)	45	Intervention: *Bacillus coagulans* probiotic daily Control: placebo daily Outcome: disease activity, inflammatory biomarkers	60 days	– Pain Scale: difference in means: 0.46 (95% CI: 0.01, 0.91); *p* = 0.046 – Painful joints: difference in means: −0.074 (95% CI: −0.81, 0.66); *p* = 0.84 – Swollen joints: difference in means: 0.011 (95% CI: −0.62, 0.64). *p* = 0.97 – ESR: difference in means: −0.054 (95% CI: −0.49, 0.38); p = 0.80 CRP: difference in means: 0.008 (95% CI: −0.52, 0.53); *p* = 0.98	Adjunctive therapy with the probiotic *Bacillus coagulans* GBI-30, 6,086 resulted in a statistically significant improvement in the patient-reported Pain Scale (*p =* 0.046) and a borderline improvement in the Patient Pain Assessment score (*p =* 0.052) compared to placebo There were no statistically significant differences between the groups in tender or swollen joint counts, HAQ disability score, or the inflammatory marker ESR. While the study’s text claims a reduction in CRP, the formal statistical comparison between groups was not significant	+
de Los Angeles Pineda et al. (54)	29	Intervention: probiotic *L. rhamnosus* GR-1 and *L. reuteri* RC-14 capsules Control: placebo Outcome: disease activity	3 months	ACR20 response: – Probiotic group: 20% (3 subjects) met the criteria – Placebo group: 7% (1 subject) met the criteria; *p* = 0.33 DAS – Mean change from baseline to final visit – Probiotic group: −2.1 ± 1.1 – Placebo group: −2.9 ± 0.6 – Comparison between groups: *p* = 0.77 IL-1 – Probiotic: +3.0 ± 12.4, Placebo: −16.1 ± 54.7; *p* = 0.06 (favors placebo) IL-6 – Probiotic: −5.0 ± 15.1, Placebo: −16.4 ± 50.5; *p* = 0.004 (favors placebo) TNF-alpha – Probiotic: −0.2 ± 3.7, Placebo: −5.2 ± 19.8; *p* = 0.03 (favors placebo) ESR – Probiotic: −4.0 ± 9.8, Placebo: 0.27 ± 6.8; *p* = 0.76 CRP Probiotic: 1.8 ± 8.4, Placebo: 1.2 ± 4.8; *p* = 0.75	There is no discernible variation in any of the ACR20 criteria’s separate components There was a noteworthy enhancement in the probiotic group’s HAQ score between visits 1 and 3 (*p =* 0.02), without any discernible variations between the groups	+
Vaghef-Mehrabani et al. (55)	46	Intervention: *L. casei* capsule (probiotic) Control: placebo capsule (maltodextrin) Outcome: disease activity, inflammatory biomarkers	8 weeks	IL-10/IL-1β – Placebo group: baseline: 0.17 [0.04–1.09], end of study: 0.12 [0.00–0.70] – Probiotic group: baseline: 0.03 [0.00–0.24], end of study: 0.06 [0.00–0.38] IL-10/IL-6 – Placebo group: baseline: 0.09 [0.00–0.21], end of study: 0.02 [0.00–0.12] – Probiotic group: baseline: 0.04 [0.00–0.17], end of study: 0.03 [0.00–0.30] IL-10/IL-12 – Placebo group: baseline: 0.01 [0.00–0.02], end of study: 0.02 [0.00–0.02] – Probiotic group: baseline: 0.00 [0.00, 0.01], end of study: 0.00 [0.00–0.03] IL-10/TNF-α – Placebo group: baseline: 0.41 [0.06–1.25], end of study: 0.19 [0.00–1.15] – Probiotic group: baseline: 0.22 [0.00–0.55], end of study: 0.17 [0.00–2.66] IL-10/total Th1 – Placebo group: baseline: 0.01 [0.00–0.02], end of study: 0.00 [0.00–0.02] – Probiotic group: baseline: 0.00 [0.00–0.01], end of study: 0.00 [0.00–0.02] – Cytokine ratios: a significant difference was found between the probiotic and placebo groups at the end of the study for IL-10/IL-12 (*p* = 0.038) and IL-10/total Th1 (*p* = 0.006) DAS28 A significant decrease was observed in the probiotic group compared to the placebo group (*p* = 0.039)	Considerable reduction in disease activity (DAS28, *p =* 0.039) and elevation in IL-10/TNF-α, IL-10/IL-12, and IL-10/total Th1 (*p =* 0.039, *p =* 0.012, and *p =* 0.014, correspondingly) Upon completion of the investigation, a noteworthy distinction was seen between the two cohorts concerning IL-10/IL-12 and IL-10/total Th1 (*p =* 0.038 and *p =* 0.006, respectively)	+
Zamani et al. (56)	60	Intervention: probiotic capsule Control: placebo Outcome: disease activity and inflammatory biomarkers	8 weeks	DAS28 – Placebo: baseline: 4.1 ± 0.7, end of trial: 4.0 ± 0.7, change: −0.1 ± 0.4; *p* = 0.31 – Probiotic: baseline: 4.0 ± 0.7, end of trial: 3.7 ± 0.7, change: −0.3 ± 0.4; *p* < 0.001 – Comparison between groups: *p* = 0.01 hs-CRP – Placebo: baseline: 6.02 ± 5.78, end of trial: 9.09 ± 7.46, change: 3.07 ± 5.53; *p* = 0.001 – Probiotic: baseline: 7.27 ± 6.24, end of trial: 6.61 ± 6.03, change: −0.66 ± 2.56; *p* = 0.25 Comparison between groups: p < 0.001	Supplementing with probiotics enhanced DAS28 (0.3 vs. 0.1 vs. 0.4, *p =* 0.01) Probiotic supplementation resulted in a significant decrease in serum insulin levels (2.0 ± 4.3 vs. +0.5 ± 4.9 lIU/mL, *p =* 0.03), serum hs-CRP concentrations (6.66 ± 2.56 vs. +3.07 ± 5.53 mg/L, *p* < 0.001), and homeostatic model assessment-B cell function (HOMA-B) (7.5 ± 18.0 vs. +4.3 ± 25.0, *p =* 0.03) When compared to the placebo, subjects who took probiotic capsules saw a marginally statistically significant improvement in their total- and low-density lipoprotein cholesterol levels (*p =* 0.09) and (*p =* 0.07)	+
Vadell et al. (59)	50	Intervention: diet containing a portfolio of suggested anti-inflammatory foods Control: diet similar to the general dietary intake in Sweden Outcome: disease activity	11 months	DAS28-ESR Mean change: – Intervention: −0.369 (95% CI: −0.628, −0.111) – Control: −0.080 (95% CI: −0.335, 0.174) – Effect size (mean difference): −0.289 (95% CI: −0.652, 0.075); *p* = 0.116 DAS28-CRP Mean change: – Intervention: −0.455 (95% CI: −0.698, −0.212) – Control: −0.222 (95% CI: −0.461, 0.017) – Effect size (mean difference): −0.233 (95% CI: −0.569, 0.103); *p* = 0.169 ESR Mean change, transformed values: – Intervention: −0.051 (−0.347–0.245) – Control: 0.210 (−0.081–0.501) Effect size (mean difference): −0.261 (−0.661–0.138); *p* = 0.194	There was no significant difference in DAS28-ESR between the intervention and control periods (*p =* 0.116) in the primary analysis, which used a linear mixed ANCOVA model with the 47 patients who had completed at least one diet session DAS28-ESR significantly decreased during the intervention period and was significantly lower after the intervention than after the control period in participants who completed both diets (*n* = 44; median 3.05; IQR: 2.41, 3.79 compared to median: 3.27; IQR: 2.69, 4.28; *p =* 0.04, Wilcoxon’s signed rank test)	+
Raad et al. (61)	44	Intervention: Mediterranean Diet (MedDiet), three video teleconsultations and two follow-up telephone calls facilitated by a registered dietitian Control: Irish Healthy Eating Guidelines (HEG) Outcome: quality of life and physical function	12 weeks	HAQ-DI – MedDiet: baseline: 0.9 ± 0.5, end of trial: 0.5 ± 0.4, change: −0.3 ± 0.3 – HEG: baseline: 1.4 ± 0.7, end of trial: 1.0 ± 0.6, change: −0.4 ± 0.4 – Comparison within group: MedDiet: *p* < 0.00, HEG: *p* < 0.001 – Comparison between groups for change: *p* = 0.586 HAQ-DI pain – MedDiet: baseline: 40.3 ± 27.5, end of trial: 17.4 ± 22.2, change: −22.9 ± 21.4 – HEG: baseline: 45.0 ± 24.3, end of trial: 30.3 ± 30.1, change: −14.7 ± 33.1 – Comparison within group: MedDiet: *p* < 0.001, HEG: *p* = 0.028 Comparison between groups for change: *p* = 0.363	Participants in the MedDiet group reported significantly better final scores for physical function (HAQ-DI, *p =* 0.006) and quality of life (*p =* 0.037) compared to the HEG group Both dietary groups demonstrated significant improvements from their own baselines in physical function, quality of life, and patient-perceived pain	+
Adam et al. (60)	68	Group 1: western diet (WD) Group 2: anti-inflammatory diet (AID) Intervention: placebo or fish oil capsules (30 mg/kg body weight) Outcome: disease activity and inflammatory biomarkers	8 months	TJC Fish oil period: – AID group: experienced a 28% reduction – WD group: experienced an 11% reduction – Comparison between groups for fish oil effect: *p* < 0.01 SJC Fish oil period: – AID group: experienced a 34% reduction – WD group: experienced a 22% reduction – *p*-value (between groups for fish oil effect): *p* < 0.01 CRP Fish oil period only – AID group: baseline: 1.6 ± 1.5, end of trial: 1.5 ± 1.6 – WD group: baseline: 2.2 ± 2.5, end of trial: 2.4 ± 2.9 – Comparison between groups for fish oil effect: *p* < 0.05 – The text states that fish oil reduced CRP in AID patients but not in WD patients. Data for the placebo period was not reported ESR Fish oil period only – AID group: baseline: 23.9 ± 16.6, end of trial: 24.4 ± 22.7 – WD group: baseline: 25.7 ± 13.2, end of trial: 25.3 ± 15.1 – The paper states that ESR was not influenced by fish oil in either group. Data for the placebo period was not reported LTB4 Fish oil period only – AID group: showed a significant decrease from baseline (*p* = 0.009) WD group: showed a significant decrease only when fish oil was given in the later phase (months 6–8), but not in the early phase	Patients in both groups were assigned to receive placebo or fish oil capsules for 3 months with a 2-month washout period between treatments During placebo treatment, the number of sore and swollen joints decreased by 14% in AID patients but not in WD patients Fish oil significantly reduced the proportion of tender (28% vs. 11%) and swollen (34% vs. 22%) joints in AID patients compared to WD patients (*p* < 0.01) AID patients had decreased production of leukotriene B4 (34% vs. 8%, *p* > 0.01), 11-dehydro-thromboxane B2 (15% vs. 10%, *p* < 0.05), and prostaglandin metabolites (21% vs. 16%, *p* < 0.003), and higher enrichment of eicosapentaenoic acid in erythrocyte lipids (244% vs. 217%)	+
Elkan et al. (64)	66	Intervention: vegan gluten free diet Control: non-vegan diet Outcome: blood lipids, inflammatory biomarkers, disease activity	12 months	DAS28 – Vegan: baseline 5.3 (5.0–5.7) → 3 months 4.7 (4.3–5.2, p = 0.002) → 12 months 4.3 (3.8–4.9, *p* < 0.001) – Non-vegan: baseline 5.3 (4.9–5.6) → 3 months 5.0 (4.6–5.3, *p* = 0.014) → 12 months 5.0 (4.6–5.4) HAQ – Vegan: baseline 1.4 (1.2–1.5) → 3 months 1.1 (0.9–1.3, *p* = 0.010) → 12 months 1.0 (0.8–1.2, p = 0.001) – Non-vegan: baseline 1.3 (1.1–1.5) → 3 months 1.2 (1.0–1.4) → 12 months 1.2 (1.0–1.4) CRP – Vegan: baseline 13 (6–26) → 3 months 11 (5–29) → 12 months 5 (4–20, *p* = 0.008) – Non-vegan: baseline 22 (5–32) → 3 months 10 (5–33) → 12 months 12 (4–19) Direct comparison between groups at 12 months DAS28: the vegan group had significantly lower disease activity than the non-vegan group (*p* = 0.047) HAQ: there was no statistically significant difference in physical function between the two groups CRP: a direct statistical comparison for CRP levels between the groups was not reported, but only the vegan group showed a statistically significant reduction from their own baseline	Compared to the control diet, the gluten-free vegan diet resulted in lower BMI, lower density lipoprotein (LDL), and increased anti-PC IgM (*p* < 0.005) After 3 months and 12 months (*p* < 0.01), the vegan group showed a drop in BMI, LDL, and cholesterol. Additionally, after 3 months (*p =* 0.021) and 12 months (*p =* 0.090), there was a trend in oxLDL levels After 3 months (*p =* 0.027), there was a trend increase in IgA anti-PC levels and a trend increase in IgM anti-PC levels after 12 months (*p =* 0.057) When compared to baseline and CRP at 12 months, the vegan group’s DAS28 and HAQ scores were considerably lower at 12 months, (respectively *p* < 0.001 and *p =* 0.001) DAS28 in the non-vegan diet group decreased significantly at 3 months but not at 12 months (*p =* 0.19), while the CRP and HAQ scores remained constant over time	+
Ghaseminasab-Parizi et al. (66)	120	Intervention 1: flaxseed (30 g/day) plus anti-inflammatory diet (AIF group) Intervention 2: flaxseed (30 g/day) plus regular diet (RF group) Control: roasted wheat (30 g/day) plus regular diet (RW group) Outcome: disease activity and inflammatory biomarkers	12 weeks	DAS28-ESR AIF group: – Baseline: 3.61 ± 1.20 – End of study: 3.13 ± 0.97 – Change within group: −0.48 ± 0.93; *p* = 0.057 RF group: – Baseline: 3.80 ± 1.13 – End of study: 2.93 ± 1.04 – Change within group: −0.87 ± 1.11; *p* = 0.001 RW group (control): – Baseline: 2.63 ± 0.86 – End of study: 2.87 ± 1.09 – Change within group: +0.24 ± 0.78; *p* = 0.110 – Between-group comparison: *p* = 0.024 CRP AIF group: – Baseline: 20.4 ± 19.3 – End of study: 17.2 ± 23.2 – Change within group: −3.2 ± 24.4; *p* = 0.517 RF group: – Baseline: 14.1 ± 10.4 – End of study: 13.3 ± 9.7 – Change within group: −0.81 ± 11.4; *p* = 0.712 RW group (control): – Baseline: 18.0 ± 20.2 – End of study: 14.5 ± 10.3 – Change within group: −3.5 ± 14.0; *p* = 0.234 – Between-group comparison: *p* = 0.863 ESR AIF group: – Baseline: 23.8 ± 23.1 – End of study: 23.7 ± 23.5 – Change within group: −0.12 ± 19.9; *p* = 0.976 RF group: – Baseline: 25.0 ± 11.4 – End of study: 20.3 ± 8.4 – Change within group: −4.7 ± 9.6; *p* = 0.018 RW group (control): – Baseline: 20.2 ± 14.6 – End of study: 15.5 ± 9.8 – Change within group: −4.7 ± 9.7; *p* = 0.025 Between-group comparison: *p* = 0.247	In the RF group, flaxseed reduced DAS28 in comparison to the RW group (−0.87 ± 1.11 vs. −0.24 ± 0.78; *p =* 0.014) In the AIF and RF groups, there was a substantial decrease in pain severity (*p* ≤ 0.001), morning stiffness (*p* < 0.05), and disease feeling (*p* < 0.01) All three groups saw improvements in HAQ disability and quality of life measures; however, AIF and RF groups saw the most improvements (*p* < 0.001) when compared to RW	+
Hafström et al. (65)	66	Intervention: vegan diet free of gluten Control: non-vegan diet Outcome: disease activity	1 year	ACR20 response rate The ACR20 criteria measure a 20% improvement in RA signs and symptoms. The response rate was significantly higher in the vegan group Valid compliant completers: patients who followed the diet for at least 9 months – Vegan diet group: 40.5% achieved ACR20 response at 12 months – Non-vegan diet group: 4.0% (1 patient) achieved ACR20 response at 12 months Intention-to-treat: all randomized patients who began the diet – Vegan diet group: 34.3% achieved ACR20 response at 12 months – Non-vegan diet group: 3.8% achieved ACR20 response at 12 months CRP When analyzing the entire vegan group, there was no statistically significant improvement in CRP. However, within the subgroup of responders to the vegan diet, CRP levels improved significantly: Baseline: 24.9 ± 31.3; 12 months: 11.8 ± 16.0; *p* < 0.05	4% of the non-vegan group and 40.5% of the vegan group met the ACR20 improvement criteria (*p* < 0.05) The patients treated with a vegan diet showed a drop in IgG levels against gliadin (*p =* 0.0071) and B-lactoglobulin (*p =* 0.0105) in the responder subgroup, but not in the other groups analyzed	+
Hartmann et al. (67)	53	Intervention: a 7- day fast followed by an 11-week PBD Control: 12-week standard DGE diet Outcome: disease activity, cardiovascular risk factors, quality of life	12 weeks	DAS28-CRP Fasting + PBD group: – Baseline: 3.89 ± 1.26 – Change at week 12: −0.97 ± 0.96 DGE group (control): – Baseline: 4.03 ± 1.39 – Change at week 12: −1.14 ± 1.10 – Between-group comparison at week 12: *p*-value = 0.568 DAS28-ESR Fasting + PBD group: – Baseline: 4.19 ± 1.41 – Change at week 12: −0.99 ± 1.09 DGE group (control): – Baseline: 4.42 ± 1.58 – Change at week 12: −1.13 ± 1.24 – Between-group comparison at week 12: *p*-value = 0.683 CRP Fasting + PBD group: – Baseline: 2.84 ± 3.54 – Change at week 12: −0.66 ± 3.17 DGE group (control): – Baseline: 3.36 ± 4.01 – Change at Week 12: 4.55 ± 0.91 – Between-group comparison at week 12 (p-value): 0.423 ESR Fasting + PBD group: – Baseline: 15.08 ± 12.45 – Change at week 12: −2.30 ± 7.87 DGE group (control): – Baseline: 16.71 ± 11.77 – Change at week 12: 2.36 ± 13.82 Between-group comparison at week 12: *p*-value = 0.162	While the DGE group improved later at 6 and 12 weeks (1 to 0.23, *p =* 0.032), the HAQ-DI improved quickly in the fasting group by day 7 and remained steady over 12 weeks (1 to 0.29, *p =* 0.001) By week 12, DAS28 had improved in both groups (1–0.97, *p* < 0.001 and 1–1.14, *p* < 0.001, respectively), with three patients in the DGE group and nine patients in the fasting group reaching ACR50 or higher CV risk variables, such as weight, improved more in the fasting group (1–3.9 kg, *p* < 0.001 and 1 to 0.7 kg, *p =* 0.146) than in the DGE group	+
Holst-Jensen et al. (68)	30	Intervention: peptide diet Control: usual food Outcome: pain intensity, disease activity	6 months	Ritchie articular index Diet group: – Baseline: 9.5 (4.0/21.5) – 4 weeks: 9.5 (3.9/27.9) – 3 months: 11.5 (3.9/24.8) – 6 months: 10.0 (5.3/16.4) – Within-group changes: no significant changes from baseline at any time point Control group: – Baseline: 12.5 (7.3/33.0) – 4 weeks: 11.5 (4.6/32.2) – 3 months: 12.0 (3.3/32.1) – 6 months: 10.0 (3.6/23.0), – Within-group changes: *p* < 0.05 – Between-group comparison: no significant difference between groups at any time point ESR Diet group: – Baseline: 34 (14/66) – 4 weeks: 22 (12/80) – 3 months: 37 (16/87) – 6 months: 40 (19/93) – Within-group changes: no significant changes from baseline at any time point Control group – Baseline: 46 (19/99) – 4 weeks: 53 (13/112) – 3 months: 55 (11/115) – 6 months: 47 (6/121) – Within-group changes: no significant changes from baseline at any time point – Between-group comparison: at 4 weeks, the diet group had a significantly lower ESR (*p* = 0.018) CRP Diet group: – Baseline: 11 (5/57) – 4 weeks: 8 (5/95) – 3 months: 9 (5/86) – 6 months: 11 (4/59) Control group: – Baseline: 25 (10/78) – 4 weeks: 23 (5/90) – 3 months: 20 (5/89) – 6 months: 15 (4/142) Within-group & between-group comparison: no statistically significant changes were found for CRP	The diet resulted in transient but statistically significant improvements in pain (*p =* 0.02) and HAQ-score (*p =* 0.03) at the 4-week mark. These improvements, however, disappeared by the 3-month follow-up after patients returned to their normal food intake Although the diet group showed significantly lower ESR and number of swollen joints immediately after the intervention, these changes were not statistically significant when analyzed for group differences. No significant changes were found in CRP levels	+
Sadeghi et al. (62)	154	Intervention 1: Mediterranean diet (MD) Intervention 2: low-fat high-carbohydrate diet Control: regular diet Outcome: disease activity	12 weeks	DAS 28 MD group: – Baseline: 3.6 ± 0.92 – End of study: 2.0 ± 1.1 – Mean change: −1.5 ± 3.01 LF-HC group: – Baseline: 3.5 ± 0.88 – End of study: 2.67 ± 1.05 – Mean change: −0.84 ± 0.98 Control group: – Baseline: 3.8 ± 0.91 – End of study: 2.9 ± 1.05 – Mean change: −0.88 ± 0.86 – Between-group comparison of change from baseline: the change in the MD group was significantly greater than in the LF-HC group (*p* = 0.02) and the control group (*p* = 0.001) – Between-group comparison of final scores: *p* < 0.001 ESR MD group: – Baseline: 19.7 ± 11.6 – End of study: 9.23 ± 10.3 – Mean change: −8.5 ± 5.6 LF-HC group: – Baseline: 21.3 ± 18.03 – End of study: 16.87 ± 13.7 – Mean change: −4.4 ± 7.9 Control group: – Baseline: 25.3 ± 16.9 – End of study: 24.66 ± 16.4 – Mean change: −0.65 ± 2.4 – Between-group comparison of final scores: *p* < 0.001 Between-group comparison of change from baseline: *p* < 0.001	Regardless of weight reduction, the MD had better effects on DAS 28 in RA patients (*p =* 0.02) than the LF-HC diet and to the control group (*p =* 0.001) The reduction in ESR was significantly greater in the MD group compared to both the LF-HC and control groups. The reduction was also significantly greater in the LF-HC group compared to the control group	+
Sköldstam et al. (63)	56	Intervention: Mediterranean diet (MD) Control: normal Western diet Outcome: disease activity	12 weeks	DAS28 – Between-group comparison of change (*p*-value): 0.047 MD group: – Baseline: 4.4 ± 1.2 – Week 3: — – Week 6: 4.2 ± 1.4 – Week 12: 3.9 ± 1.2 (*p* < 0.001 vs. baseline) – Week 12 change: −0.56 (*p* < 0.001 vs. baseline, as per abstract) Control group: – Baseline: 4.3 ± 1.4 – Week 3: — – Week 6: 4.2 ± 1.4 – Week 12: 4.3 ± 1.5 – Between-group comparison of change: *p* = 0.047 CRP – Between-group comparison of change (*p*-value): 0.006 MD group: – Baseline: 17 ± 20 – Week 3: 16 ± 22 – Week 6: 27 ± 55 – Week 12: 12 ± 15 (*p* = 0.001 vs. baseline) – Control group: showed no significant changes from baseline at any time point – Baseline: 15 ± 14 – Week 3: 15 ± 16 – Week 6: 12 ± 9 – Week 12: 15 ± 12 ESR MD group: – Baseline: 24 ± 15 – Week 3: 28 ± 20 – Week 6: 31 ± 23 (*p* = 0.027 vs. baseline) – Week 12: 25 ± 15 Control group: – Baseline: 23 ± 15 – Week 3: 26 ± 20 – Week 6: 22 ± 15 – Week 12: 25 ± 19 Between-group comparison of change: *p* = 0.660	During the study, patients in the MD group (*n* = 26) experienced a decrease in DAS28 of 0.56 (*p* < 0.001), a decrease in HAQ of 0.15 (*p =* 0.020), and an increase in “vitality” of 11.3 (*p =* 0.018) and a decrease in “compared with 1 year earlier” of 0.6 (*p =* 0.016) on the SF-36 Health Survey At the conclusion of the research, there was no discernible change for the control patients (*n* = 25)	+
Sundrarjun et al. (39)	60	Intervention 1: low n-6 fatty acid diet supplemented with fish oil Intervention 2: low n-6 fatty acid diet supplemented with placebo Control: no special diet Outcome: inflammatory biomarkers	24 weeks	SJC Fish oil group: – Baseline (week 0): 8.60 ± 1.02 – Week 6: 10.26 ± 1.27 – Week 18: 8.78 ± 1.23 – Week 24: 7.69 ± 1.34 Placebo group: – Baseline: 10.13 ± 1.56 – Week 6: 11.52 ± 1.73 – Week 18: 9.47 ± 1.51 – Week 24: 8.52 ± 1.48 Control group: – Baseline: 10.10 ± 1.34 – Week 6: 7.10 ± 1.52 – Week 18: 8.60 ± 1.11 – Week 24: 6.70 ± 0.98 – Between-group comparison: no significant difference between groups TJC Fish oil group: – Baseline: 11.56 ± 1.96 – Week 6: 11.13 ± 1.76 – Week 18: 9.13 ± 1.38 – Week 24: 8.82 ± 1.36 Placebo group: – Baseline: 10.39 ± 2.10 – Week 6: 11.43 ± 2.13 – Week 18: 12.30 ± 2.15 – Week 24: 10.86 ± 2.19 Control group: – Baseline: 14.20 ± 2.82 – Week 6: 9.50 ± 2.21 – Week 18: 8.80 ± 2.50 – Week 24: 6.90 ± 2.23 – Between-group comparison: no significant difference between groups	ESR Compared to baseline, the fish oil group showed significant increases in docosahexaenoic acid and eicosapentaenoic acid (*p* < 0.05) and significant decreases in linoleic acid (*p* < 0.05), CRP (*p* < 0.05), and sTNF-R p55 (*p* < 0.05) Interleukin-6 and TNF-a significantly decreased in the fish oil and placebo groups by week 24 Patients with RA experienced a decrease in serum levels of sTNF-R p55 and CRP when they were supplemented with n-3 FA and consumed less n-6 FA	∅
				Fish oil group: – Baseline: 73.34 ± 5.77 – Week 6: 66.04 ± 6.45 – Week 18: 67.60 ± 7.29 – Week 24: 63.30 ± 6.95 Placebo group: – Baseline: 68.47 ± 7.30 – Week 6: 64.60 ± 6.82 – Week 18: 56.60 ± 6.90 – Week 24: 59.26 ± 6.73 Control group: – Baseline: 57.00 ± 7.19 – Week 6: 56.57 ± 8.38 – Week 18: 54.71 ± 8.49 – Week 24: 50.78 ± 8.96 – Between-group comparison: no significant difference between groups CRP Fish oil group: – Baseline: 51.12 ± 9.13 – Week 6: 46.18 ± 9.44 – Week 18: 37.27 ± 8.70 – Week 24: 34.65 ± 8.27 Placebo group: – Baseline: 29.15 ± 5.63 – Week 6: 28.21 ± 5.96 – Week 18: 25.81 ± 6.49 – Week 24: 21.34 ± 5.98 Control group: – Baseline: 40.39 ± 11.0 – Week 6: 36.24 ± 10.94 – Week 18: 38.59 ± 10.68 – Week 24: 40.60 ± 11.89 Statistically significant reduction from baseline (*p* < 0.05) within the fish oil group only at week 18 and 24		
Kremer et al. (41)	44	Intervention: diet high in polyunsaturated fat and low saturated fat + eicosapentaenoic acid supplement Control: diet lower polyunsaturated to saturated ratio + placebo supplement Outcome: disease activity	12 weeks	Change from baseline while taking NSAIDs (weeks 0 to 18/22) Fish oil group TJC: – Mean change: −5.3 ± 0.835; *p* < 0.0001 Corn oil group: – The article states that none of the changes from baseline achieved statistical significance. It only provides a specific value for the trend in swollen joint count SJC: – Mean change: −1.3 ± 0.68; *p* = 0.06 Overall change from baseline after discontinuing NSAIDs (weeks 0 to 26/30) Fish oil group TJC: – Mean change: −7.8 ± 2.6; *p*-value: 0.011 – This change was statistically significant compared to the change in the corn oil group (*p* = 0.043) SJC: – Mean change: −4.7 ± 2.7; *p* = 0.10 Corn oil group: TJC: – Mean change: −6.4 ± 2.2; *p* = 0.78 SJC: – Mean change: −5.6 ± 1.7; *p* = 0.004 IL-1β in fish oil group Change from baseline to week 18/22: – Mean change: −7.7 ± 3.1; *p* = 0.026 TNF-α Change from baseline to week 26/30 (fish oil group): – Mean change: +45.1 ± 13.6; *p* = 0.013 Change from baseline to week 26/30 (corn oil group): – Mean change: +65.8 ± 27.5; *p* = 0.038 IL-6 No inhibitory effect of fish oil was demonstrated; no significant changes were reported for IL-6	While patients were also taking the NSAID diclofenac, the fish oil group experienced statistically significant improvements from baseline in several key areas: number of tender joints (*p* < 0.0001), duration of morning stiffness (*p =* 0.008), physician’s and patient’s global evaluation of arthritis activity (*p =* 0.017 and *p =* 0.036, respectively), physician’s evaluation of pain (*p =* 0.004). In contrast, the corn oil group showed no significant improvements in any clinical parameters during this period After discontinuing the NSAID, the benefits of fish oil persisted. The reduction in tender joints in the fish oil group remained statistically significant compared to their baseline (*p =* 0.011). This improvement in tender joints was significantly greater than the change seen in the corn oil group (*p =* 0.043), demonstrating a clear advantage for fish oil in this phase The clinical improvements were associated with a significant decrease in serum levels of the pro-inflammatory cytokine interleukin-1β in the fish oil group (*p =* 0.026). The study did not find a significant inhibitory effect on other measured cytokines like IL-2, IL-6, IL-8, or TNF-α	+
Magaro et al. (42)	12	Intervention: diet high in polyunsaturated fatty acids supplemented with eicosapentaenoic and docosahexaenoic acids Control: isoenergetic diet Outcome: disease activity and inflammatory biomarkers	1 month	Ritchie’s articular index Group B (fish oil): – Baseline: 17.2 ± 3.38 – 30 days: 10.6 ± 3.48 – *p* < 0.01 – Group A (control): showed no significant change – Baseline value not explicitly stated but was not significantly different from Group B’s baseline – 30 Days: 21.4 ± 3.2 – Between-group comparison at 30 Days: the score was significantly lower in the fish oil group (*p* < 0.005) ESR – Group B (fish oil): showed a statistically significant improvement (*p* < 0.01), as indicated by the asterisk in figure of the article – Group A (control): showed no significant change, data shown in figure Between-group comparison: the text states there were “no statistically significant changes” between the two dietary regimens for ESR	Regarding neutrophil chemiluminescence and clinical indicators, there was no statistically significant variation seen among the patients treated with a diet high in saturated fatty acids Eating fish oil reduced neutrophil chemiluminescence and subjectively relieved active rheumatoid arthritis	∅

This table summarizes the risk of bias assessment for each included study based on predefined quality criteria. Studies were categorized as “Positive” if they met >80% of criteria, “Neutral” if they met 50–80%, and “Negative” if they met <50%. RCT, randomized controlled trial; DAS28-ESR, Disease Activity Score 28-Erythrocyte Sedimentation Rate; FoxP3, Forkhead Box P3; RORγt, RAR-related orphan receptor-γ; PPAR-γ, peroxisome proliferator-activated receptor gamma; CRP, C-reactive protein; IL-6, interleukin-6; MedDiet/MD, Mediterranean diet; hs-CRP, high-sensitivity C-reactive protein; VAS, Visual Analog Scale; NO, nitric oxide; HOMA-IR, homeostatic model assessment for insulin resistance; HOMA-B, homeostatic model assessment for beta cell function; GSH, glutathione; POMx, pomegranate extract; HAQ, Health Assessment Questionnaire; GPx, glutathione peroxidase; NAC, N-acetylcysteine; MDA, malondialdehyde; FBS, fasting blood sugar; LDL-C, low-density lipoprotein cholesterol; HDL-C, high-density lipoprotein cholesterol; dPP, plant-derived polysaccharide; ω-3 FA, omega-3 fatty acids; DHA, docosahexaenoic acid; n-3 LC-PUFA, n-3 long-chain polyunsaterated fatty acid; BMI, body mass index; IgA/M anti-PC, immunoglobulin A/M against phosphorylcholine; ACR, American College of Rheumatology; PBD, plant-based diet; DGE, Deutsche Gesellschaft fürErnährung; LGG, *Lactobacillus rhamnosus* GG; RF, rheumatoid factor; ESR, erythrocyte sedimentation rate; LD-1227, peptide-rich marine biology formula; EPA, eicosapentaenoic acid; DHA, docosahexaenoic acid; GLA, gamma-linolenic acid; sTNF-R p55, soluble tumour necrosis factor receptor p55.

**Table 2:** Key features of the included studies, subdivided according to chronic arthritis subtype—spondyloarthritis.

Study	Sample size (*n*)	Interventions, controls and outcome	Duration	Reported results	Main findings	Quality assessment rating
Sundström et al. (72)	24	Intervention: high-dose (4.55 g omega-3/day) supplement Control: low-dose (1.95 g omega-3/day) supplement Outcome: disease activity	21 weeks	BASDAI High-dose group: – Baseline: 4.32 ± 1.32 – Week 7: 2.62 ± 1.92 – Week 14: 3.37 ± 1.12 – Week 21: 2.92 ± 1.42 – *p*-value for change (baseline vs. week 21): 0.038 Low-dose group: – Baseline: 3.01 ± 2.39 – Week 7: 3.00 ± 1.58 – Week 14: 2.72 ± 2.15 – Week 21: 2.44 ± 2.99 – *p*-value for change (baseline vs. week 21): 0.859 ESR High-dose group: – Baseline: 10 ± 14 – Week 7: 8 ± 14 – Week 14: 10 ± 16 – Week 21: 10 ± 16 – *p*-value for change (baseline vs. week 21): 0.859 Low-dose group: – Baseline: 21 ± 14 – Week 7: 20 ± 18 – Week 14: 30 ± 24 – Week 21: 26 ± 29 *p*-value for change (baseline vs. week 21): 0.027	The high-dose group (4.55 g/day) showed a statistically significant decrease in disease activity as measured by the BASDAI from baseline to the end of the 21-week study (*p* = 0.038). In contrast, the low-dose group (1.95 g/day) did not show a significant change in BASDAI Interestingly, the low-dose group experienced a statistically significant increase in ESR (*p* = 0.027), while the high-dose group did not There were no significant differences in functional capacity (BASFI) in either group, and no significant differences were found when directly comparing the final results between the high- and low-dose groups	+
Jenks et al. (69)	63	Intervention: oral probiotic Control: placebo Outcome: disease activity, quality of life	12 weeks	BASDAI Probiotic group: – Baseline: 4.2 ± 2.2 – Week 12: 3.2 ± 2.1 – Probiotic effect (95% CI): −0.6 (−1.6 to 0.3). While the probiotic group improved slightly more, the difference was not statistically significant. *p* = 0.182 Placebo group: – Baseline: 4.5 ± 2.0 – Week 12: 3.9 ± 2.2 CRP Probiotic group: – Baseline: 6.8 ± 6.7 – Week 12: 6.7 ± 6.3 – Probiotic effect (95% CI): −3.5 (−7.8 to 0.8). While the mean change favored the probiotic group, the difference was not statistically significant. Placebo group: – Baseline: 10.0 ± 11.3 – Week 12: 11.3 ± 11.2 Between-group comparison: no data	The probiotic group’s mean BASFI decreased from 3.5 ± 2.0 to 2.9 ± 1.9, while the placebo group’s mean BASFI decreased from 3.6 ± 1.9 to 3.1 ± 2.2 (*p* = 0.839) The probiotic group’s mean BASDAI decreased from 4.2 ± 2.2 to 3.2 ± 2.1, while the placebo group’s mean BASDAI decreased from 4.5 ± 2.0 to 3.9 ± 2.2 (*p* = 0.182)	+
Ahangari Maleki et al. (70)	48	Intervention: one synbiotic capsule daily Control: placebo daily Outcome: immune response	12 weeks	BASDAI Synbiotic group: – Baseline: 2.65 ± 1.91 – After 12 weeks: 2.51 ± 1.88 – Within-group change *p*-value: 0.744 Placebo group: – Baseline: 3.51 ± 1.75 – After 12 weeks: 3.21 ± 1.44 – Within-group change: *p* = 0.431 – Between-group comparison at 12 weeks: *p* = 0.686 ASDAS-CRP Synbiotic group: – Baseline: 2.48 ± 0.96 – After 12 weeks: 2.35 ± 1.03 – Within-group change: *p* = 0.472 Placebo group: – Baseline: 2.76 ± 0.89 – After 12 weeks: 2.64 ± 0.99 – Within-group change: *p* = 0.503 – Between-group comparison at 12 weeks: *p* = 0.903 Serum IL-17 Synbiotic group: – Baseline: 38.22 ± 14.40 – After 12 weeks: 24.38 ± 11.68 – Within-group change *p*-value: 0.002 Placebo group: – Baseline: 39.16 ± 15.20 – After 12 weeks: 33.27 ± 12.84 – Within-group change: *p* = 0.188 – Between-group comparison at 12 weeks: *p* = 0.057 Serum IL-23 Synbiotic group: – Baseline: 51.77 ± 17.40 – After 12 weeks: 32.16 ± 12.46 – Within-group change: *p* < 0.001 Placebo group: – Baseline: 46.88 ± 14.68 – After 12 weeks: 41.16 ± 14.63 – Within-group change: *p* = 0.100 Between-group comparison at 12 weeks: *p* = 0.060	Compared to baseline, the proportion of IL17-expressing CD4^+^ T cells was significantly lower with synbiotic supplementation (4.88 ± 2.47 vs. 2.16 ± 1.25), as well as the gene expression of IL-17 (1.03 ± 0.24 vs. 0.65 ± 0.26), IL-23 (1.01 ± 0.13 vs. 0.68 ± 0.24), serum IL-17 (38.22 ± 14.40 vs. 24.38 ± 11.68), and IL-23 (51.77 ± 17.40 vs. 32.16 ± 12.46) Significant variations were seen solely in the percentage of CD4^+^ T cells expressing IL-17 and in the expression of the IL-17 and IL-23 genes between the groups (*p* < 0.001)	+
Brophy et al. (71)	147	Intervention: probiotic capsule (10 g lyophilized powder containing live bacteria) daily Control: placebo daily Outcome: disease activity	3 months	Disease activity (0–10 scale) Probiotic group: – Baseline: 4.1 ± 2.2 – Final: 3.6 ± 2.6 Placebo group: – Baseline: 3.5 ± 1.9 – Final: 2.9 ± 2.2 – Estimated probiotic effect (95% CI): 0.20 (−0.47 to 0.86) The result is not statistically significant	There was no significant difference, either statistically or clinically, observed in the overall well-being, gastrointestinal symptoms, or arthritis severity between the probiotic and placebo groups	∅

This table summarizes the risk of bias assessment for each included study based on predefined quality criteria. Studies were categorized as “Positive” if they met >80% of criteria, “Neutral” if they met 50–80%, and “Negative” if they met <50%. RCT, randomized controlled trial; BASFI, Bath Ankylosing Spondylitis Functional Index; BASDAI, Bath Ankylosing Spondylitis Disease Activity Index; CD, cluster of differentiation; IL, interleukin.

**Table 3:** Key features of the included studies, subdivided according to chronic arthritis subtype—psoriatic arthritis.

Study	Sample size (*n*)	Interventions, controls and outcome	Duration	Reported results	Main findings	Quality assessment rating
Kristensen et al. (73)	145	Intervention: 3 g of n-3 PUFA daily Control: olive oil daily Outcome: disease activity	24 weeks	DAS66/68 Baseline values: – n-3 PUFA group: 2.5 ± 0.9 – Control group: 2.7 ± 0.9 – Change after 24 weeks: the study reports that “Adjustment for disease activity … did not change the results,” and that disease activity “was not associated with HRV or PWV.” Specific values for the change in DAS were not provided, as this was not a primary outcome and did not change significantly CRP Baseline values: – n-3 PUFA group: 4.6 ± 4.2 – Control group: 6.1 ± 7.7 Change after 24 weeks: the study states that CRP “was not associated with heart rate variability (HRV) or pulse wave velocity (PWV).” Specific values for the change in CRP were not provided	There was a significant difference (*p* = 0.03) in the mean of all normal RR intervals between the subjects who consumed the most and the ones who consumed the least fish Patients who supplemented with n-3 PUFA showed reduced heart rate (*p* = 0.01) and increased RR (*p* = 0.01) when compared to the control group Following n-3 PUFA supplementation, there was no change in blood pressure, PWV, or central blood pressure	+
Leite et al. (74)	97	Intervention 1: diet-placebo (hypocaloric diet + placebo supplementation) Intervention 2: diet-fish (hypocaloric diet + 3 g/day of n-3 PUFA supplementation) Control: placebo Outcome: disease activity	12 weeks	DAS28-CRP Diet-fish group: – Baseline: 2.83 ± 1.55 – After 12 weeks: 2.43 ± 1.0 – Mean difference: −0.40 ± 1.11 – Comparison within-group: *p* = 0.004 Diet-placebo group: – Baseline: 2.98 ± 1.35 – After 12 weeks: 2.33 ± 1.1 – Mean difference: −0.66 ± 0.90 – Comparison within-group: *p* = 0.004 Placebo group (control): – Baseline: 2.93 ± 1.19 – After 12 weeks: 2.72 ± 1.0 – Mean difference: −0.21 ± 1.15 – Comparison within-group: *p* = 0.004 – Comparison between groups: *p* = 0.84 DAS28-ESR Diet-fish group: – Baseline: 3.31 ± 1.2 – After 12 weeks: 3.50 ± 1.4 – Mean difference: +0.19 ± 1.16 – Comparison within-group: *p* = 0.3 Diet-placebo group: – Baseline: 3.40 ± 1.6 – After 12 weeks: 2.90 ± 1.44 – Mean difference: −0.49 ± 0.89 – Comparison within-group: *p* = 0.3 Placebo group (control): – Baseline: 3.56 ± 1.3 – After 12 weeks: 3.46 ± 1.2 – Mean difference: −0.10 ± 1.4 – Comparison within-group: *p* = 0.3 – Comparison between groups: *p* = 0.52	DAS28-CRP and BASDAI values showed improvement, particularly in the Diet-placebo group (*p* = 0.004 for 0.6 ± 0.9 and *p* = 0.001 for 1.39 ± 1.97, respectively) In both intervention groups, more patients attained minimum disease activity (MDA), compared to the placebo group Significant reductions in waist circumference (−3.28 ± 3.5, *p* < 0.001), body fat (−1.2 ± 2.2, *p* = 0.006), and weight (−1.79 ± 2.4; *p* = 0.004) were seen in the diet-fish group There is no noteworthy association seen between reducing weight and improving disease activity	+
				BASDAI Diet-fish group: – Baseline: 2.51 ± 1.83 – After 12 weeks: 2.70 ± 2.40 – Mean difference: +0.19 ± 1.67 – Comparison within-group: *p* = 0.56 Diet-placebo group: – Baseline: 3.5 ± 2.23 – After 12 weeks: 2.11 ± 1.95 – Mean difference: −1.39 ± 1.97 – Comparison within-group: p = 0.001 Placebo group (control): – Baseline: 2.94 ± 1.96 – After 12 weeks: 2.31 ± 1.84 – Mean Difference: −0.63 ± 1.5 – Comparison within-group: *p* = 0.04 – Comparison between groups: *p* = 0.78		

This table summarizes the risk of bias assessment for each included study based on predefined quality criteria. Studies were categorized as “Positive” if they met >80% of criteria, “Neutral” if they met 50–80%, and “Negative” if they met <50%. RCT, randomized controlled trial; n-3 PUFA, n-3 polyunsaturated fatty acids; PWV, pulse wave velocity; Quality Assessment Rating, +, positive; ∅, neutral; −, negative; DAS29-CRP, disease activity score-29-C reactive protein; BAS AI, Bath Ankylosing Spondylitis Disease Activity Index.

### 2.6 Statistical analysis

Since no meta-analysis was performed, the results from included studies were summarized narratively, taking into account the study design, intervention characteristics, and reported outcomes. Effect sizes, confidence intervals, and *p*-values reported in the original studies were extracted. Additionally, potential sources of heterogeneity among studies were explored based on differences in methodology, population, and intervention duration.

## 3 Results

The results are presented by arthritis type (RA, axSpA, PsA) and categorized based on the type of intervention (dietary interventions, supplementation, probiotics, and synbiotics) to facilitate structured comparison.

### 3.1 Characteristics of eligible studies

**Figure 1:**
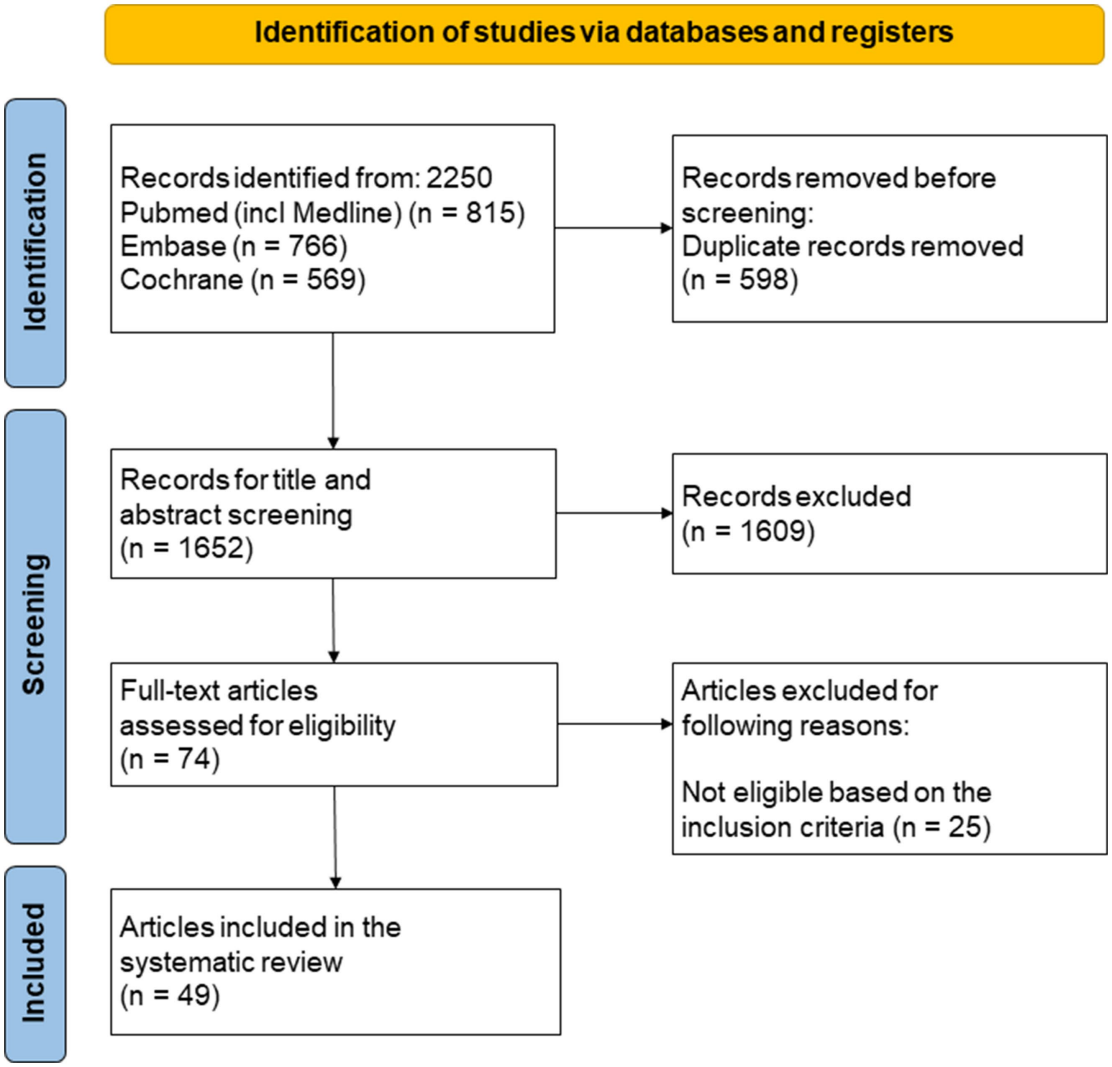
Preferred Reporting Items for Systematic Reviews and Meta-analyses (PRISMA) guidelines were followed in creating the flow diagram for screening eligible clinical trials. This diagram outlines the selection process for studies included in the systematic review. The screening process followed the PRISMA guidelines, including database searches, eligibility criteria, and reasons for exclusion at each stage.

Preferred Reporting Items for Systematic Reviews and Meta-analyses (PRISMA) guidelines were followed in creating the flow diagram for screening eligible clinical trials (Figure 1) (24). Figure 1 illustrates the stepwise selection process of eligible studies, highlighting the number of included and excluded records at each screening stage.

Searching the PubMed, Embase, and the Cochrane Library databases, 2,250 records were found. Following the removal of duplicates, 1,652 records underwent title and abstract screening, of which 1,609 were found ineligible and eliminated. Out of the 74 studies that made it through the full-text screening process, 25 studies did not meet the inclusion criteria. Forty-nine studies were selected for further discussion and quality assessment. The characteristics of included studies are summarized in Table 1 (RA studies), Table 2 (axSpA studies), and Table 3 (PsA studies). A meta-analysis was not performed due to the pervasive clinical and methodological heterogeneity across study designs, interventions, and outcomes (e.g., varying dosages, durations, and reported metrics), which prevented meaningful quantitative pooling of data. Results were therefore synthesized narratively.

The eligible studies included in the systematic review had sample sizes between 12 and 186. The main characteristics of these trials are summarized below. The duration varied from 8 weeks to 12 months. To systematically analyze the effects of nutrition on chronic arthritis, the included studies were classified according to the type of arthritis investigated. Among the studies of RA (Table 1), 24 studies explored the impacts of supplements such as ginger powder (26), polyunsaturated fatty acids (27–43), pomegranate extract (44), vitamin D (45, 46), N-acetylcysteine (47), quercetin (48), and LD-1227 (49). Additionally, nine articles concentrated on probiotics (50–56) and synbiotics (57, 58), while another 10 examined the effects of different diets, including the anti-inflammatory diet (59, 60), Mediterranean diet (61–63), vegan diet and gluten-free diet (64, 65), flaxseed diet (66), fasting regimen (67), and peptide diet (68), on RA. Regarding axSpA (Table 2), three studies (69–71) utilized probiotics as interventions, while one trial (72) supplemented with polyunsaturated fatty acids. In the case of PsA (Table 3), one article (73) investigated the impact of polyunsaturated fatty acid supplementation, while another (74) examined the effect of a hypocaloric diet supplemented with n-3 PUFA.

Thirty-seven out of 49 studies had a positive quality rating in Tables 1–3, indicating a minimal risk of bias. However, 12 studies were rated as neutral, suggesting varying degrees of bias. Less than 80% of participants in several research (28, 32, 35, 36, 39, 48, 71) were followed up with, increasing the risk of bias. The comparability between research groups was compromised in one trial (51) because the probiotic group received MTX treatment more frequently than the placebo group. In four trials (42, 43, 49, 58) dropouts were not mentioned. Incomplete data presentation was noted in one study (49). Additionally, the sample population in one trial (42) was noticeably small, which increased the risk of selection bias.

### 3.2 Effect of nutrition and diet in RA

#### 3.2.1 Diet

Various dietary interventions, including Mediterranean, anti-inflammatory, vegan, gluten-free, and flaxseed-based diets, as well as fasting and peptide diets, have been investigated for their impact on disease activity, inflammatory biomarkers, and quality of life in patients with RA.

Three studies reported a beneficial effect of the MD. Raad et al. (61) conducted a telehealth-delivered randomized controlled trial with 44 RA patients in Ireland to compare the effects of MD and the Irish Healthy Eating Guidelines (HEG) over 12 weeks. Both groups reported improvements in physical function (MD: HAQ-DI, 0.9 ± 0.5 to 0.5 ± 0.4, *p* < 0.001; HEG: 1.4 ± 0.7 to 1.0 ± 0.6, *p* < 0.001) and quality of life (MD: 10.1 ± 7.5 to 4.0 ± 4.7, *p* < 0.001; HEG: 11.25 ± 7.2 to 7.9 ± 6.4, *p* = 0.04). However, the MD group experienced significantly better outcomes in both physical function (*p* = 0.006) and quality of life (*p* = 0.03) compared to the HEG group, with increased physical activity observed only in the MD group (*p* = 0.01). Sadeghi et al. (62) evaluated the effects of MD compared to a low-fat, high-carbohydrate diet (LF-HC) and a control diet in a 12-week randomized trial involving 129 overweight and obese RA patients. The MD group showed a significant reduction in DAS28 scores compared to the LF-HC (*p* = 0.02) and control groups (*p* = 0.001), independent of weight loss. Serum ESR levels were also significantly lower in the MD group compared to the LF-HC group (*p* = 0.007) and controls (*p* < 0.001). Sköldstam et al. (63) conducted a 12-week randomized study comparing MD and a standard Western diet in RA patients with stable but active disease (*n* = 51). The MD group demonstrated significant improvements in disease activity (DAS28: −0.56, *p* < 0.001), physical function (HAQ: −0.15, *p* = 0.020), CRP levels (*p* = 0.006), and vitality scores from the SF-36 health survey (*p* = 0.018), while the control group showed no significant changes. A DAS28 reduction of this magnitude, while modest, is generally considered clinically relevant, particularly as it was accompanied by significant improvements in physical function and quality of life. The MD group also experienced weight loss (−3.0 kg, *p* < 0.001), although weight loss was not correlated with reduced disease activity.

Two trials examined the impact of anti-inflammatory diet (AID) (59, 60). Vadell et al. (59) observed a significant decrease in DAS28-ESR during an 11-month intervention involving 44 participants. Specifically, DAS28-ESR decreased significantly during the intervention period compared to baseline values (median: 3.05 vs. 3.39, *p* = 0.01) and was significantly lower after the intervention compared to the control diet period (median: 3.05 vs. 3.27, *p* = 0.04). However, in the main analysis, no statistically significant difference in DAS28-ESR was found between intervention and control diets (*p* = 0.11). This suggests that while the AID had positive effects during the intervention, its overall efficacy compared to the control diet remains inconclusive based on adjusted analyses. Adam et al. (60) conducted a crossover trial with 68 patients, comparing an AID with a low arachidonic acid intake (<90 mg/day), placebo, fish oil supplementation, and a Western diet (WD). AID alone led to a 14% reduction in tender and swollen joint counts during placebo treatment. Fish oil supplementation enhanced the effect of AID, resulting in significant reductions in tender (28%) and swollen (34%) joint counts compared to baseline (*p* < 0.01). Compared to the WD, patients on AID combined with fish oil exhibited greater increases in erythrocyte eicosapentaenoic acid levels (244% vs. 217%) and larger reductions in leukotriene B4 (34% vs. 8%, *p* < 0.01), 11-dehydro-thromboxane B2 (15% vs. 10%, *p* < 0.05), and prostaglandin metabolites (21% vs. 16%, *p* < 0.003).

Elkan et al. (64) conducted a randomized study involving 66 patients with active RA to evaluate the effects of a gluten-free vegan diet on disease activity and immune response. Participants were divided into two groups: 38 patients followed a gluten-free vegan diet, while 28 adhered to a well-balanced non-vegan diet for 1 year. Of those who completed the diet regimens for at least 9 months, 40.5% (9/22) in the vegan group achieved an ACR20 response compared to only 4% (1/25) in the non-vegan group. For the intention-to-treat population, the proportions were 34.3% (13/38) and 3.8% (1/28), respectively, demonstrating significantly higher clinical improvement in the vegan diet group, meeting accepted criteria for clinical response in RA. Immunological analysis revealed that IgG antibody levels against gliadin and β-lactoglobulin decreased in the responder subgroup of the vegan diet group but remained unchanged in non-responders and in the non-vegan group. For example, the mean IgG levels against gliadin decreased from 50 to 35 U/mL in responders, highlighting the diet’s potential role in modulating immune reactivity. Additionally, the vegan group showed significant reductions in LDL cholesterol (average decrease of 0.6 mmol/L; *p* < 0.05) and oxidized LDL levels, suggesting improved cardiovascular risk profiles. In contrast, no significant metabolic changes were observed in the non-vegan group. Radiographic analysis indicated no retardation of joint destruction in either group, implying that while the gluten-free vegan diet improved clinical and immunological outcomes, it did not influence structural joint damage over the 12-month period.

Hafström et al. (65) studied 66 patients with active RA who were randomized to either a vegan gluten-free diet (38 patients) or a well-balanced non-vegan diet (28 patients) for 1 year. Among those who completed at least 9 months on the diets (22 in the vegan group and 25 in the non-vegan group), 40.5% of the vegan diet group (9 patients) achieved the ACR20 improvement criteria compared to only 4% (1 patient) in the non-vegan group. In the intention-to-treat analysis, these figures were 34.3 and 3.8%, respectively. The vegan diet group also demonstrated reductions in IgG antibody levels against gliadin and beta-lactoglobulin, particularly in the responder subgroup, whereas no such changes were observed in the non-vegan group. However, no retardation in radiological destruction was noted in either group.

In a 12-week randomized controlled trial by Ghaseminasab-Parizi et al. (66), the effects of flaxseed consumption (30 g/day) with and without an AID were assessed in 120 patients. Participants were randomly assigned to three groups: flaxseed combined with an AID (AIF group), flaxseed with a regular diet (RF group), and roasted wheat (30 g/day) with a regular diet (RW group) as a control. Significant improvements were observed in DAS28 scores, with a reduction of −0.87 ± 1.11 in the RF group compared to −0.24 ± 0.78 in the RW group (*p* = 0.014). Both flaxseed groups (AIF and RF) experienced reductions in pain severity (*p* ≤ 0.001), morning stiffness (*p* < 0.05), and disease feeling (*p* < 0.01), as well as improved quality of life and HAQ disability index compared to the RW group (*p* < 0.001). Morning stiffness decreased significantly in the AIF and RF groups, but no significant difference between these groups was found. Physical and mental health components of quality of life, such as physical functioning, vitality, and emotional well-being, showed notable improvements in the AIF and RF groups compared to RW (*p* < 0.05). Biomarkers of inflammation, including CRP and ESR, as well as autoantibodies, showed no significant changes between groups, although rheumatoid factor levels trended toward reduction in the AIF group (*p* = 0.06).

Hartmann et al. (67) conducted a randomized controlled trial (NutriFast-Study) involving 53 RA patients to compare the effects of a 7-day fast followed by an 11-week plant-based diet (PBD) with a 12-week standard anti-inflammatory diet recommended by the German Society for Nutrition (DGE). Of the participants, 50 completed the study per protocol. Although no significant difference was observed between the two groups in the primary outcome of HAQ-DI improvement at 12 weeks (*p* = 0.66), the fasting group experienced a rapid reduction in HAQ-DI by day 7 (−0.24 ± 0.22, *p* = 0.01), sustained at 12 weeks (−0.29 ± 0.38), while the DGE group showed delayed improvements beginning at week 6 (−0.24 ± 0.49). Both groups exhibited significant reductions in DAS28 scores at 12 weeks (fasting group: −0.97 ± 0.96. DGE group: −1.14 ± 1.10, *p* < 0.001 for both), but faster responses were observed in the fasting group, where 36% achieved ACR50 or higher by week 12 compared to 12% in the DGE group, indicating a clinically meaningful response in a higher proportion of patients. Cardiovascular risk factors improved more significantly in the fasting group, including greater weight loss (−3.9 kg vs. −0.7 kg, *p* < 0.001) and reductions in LDL cholesterol and triglycerides by week 6. Holst-Jensen et al. (68) conducted a randomized controlled trial with 30 RA patients, comparing a four-week liquid peptide diet to a regular diet. The peptide diet significantly reduced pain (*p* = 0.02), HAQ scores (*p* = 0.03), and BMI (*p* = 0.001), but only one patient achieved remission.

#### 3.2.2 Supplementation

Two trials (27, 30) demonstrated a significant reduction in disease activity, measured by the DAS28 score, compared to baseline in patients with RA receiving n-3 PUFA and fish oil. In the study by Berbert et al. (29), 43 participants (34 female, 9 male) were randomly assigned into three groups: Group 1 (*n* = 13) received a placebo (soy oil), Group 2 (*n* = 13) received fish oil supplementation at a dose of 3 g/day (containing 90 mg EPA and 60 mg DHA per capsule, 20 capsules daily), and Group 3 (*n* = 17) received the same fish oil supplementation combined with 9.6 mL/day of olive oil. Significant improvements in clinical indicators such as morning stiffness duration, joint pain intensity, and handgrip strength were observed after 12 and 24 weeks, with the most pronounced effects in the group receiving both fish oil and olive oil.

Several studies (28, 30, 35, 40, 41) demonstrated reductions in swollen and tender joint counts following PUFA supplementation. In Geusens et al. (32), a 12-month, double-blind, randomized trial with 90 RA patients compared 2.6 g/day of n-3 PUFA, 1.3 g/day of n-3 PUFA plus 3 g/day of olive oil, and 6 g/day of olive oil. Only the 2.6 g/day group showed significant improvements in patient-reported outcomes and physician-assessed pain, with more patients reducing antirheumatic medications, highlighting the clinical efficacy of this dose.

Fish oil supplementation has been associated with reductions in key inflammatory markers, including ESR and CRP (27, 33). In the study by Fatel et al. (27), 62 participants (50 female, 12 male) were divided into three groups: the control group (*n* = 21), the fish oil group (*n* = 21) receiving 3 g/day of fish oil (containing 180 mg EPA and 120 mg DHA per capsule, 10 capsules daily), and the cranberry juice group (*n* = 20) receiving the same dose of fish oil combined with 500 mL/day of reduced-calorie cranberry juice. After 90 days, the fish oil group showed significant reductions in DAS28-CRP (*p* = 0.02) and adiponectin levels (*p* = 0.02). The cranberry juice group demonstrated even greater benefits, with reductions in DAS28-CRP (*p* = 0.001), ESR (16.0 to 11.0 mm/h, *p* = 0.033), and CRP (3.7 to 2.5 mg/dL, *p* = 0.002), indicating a synergistic effect of fish oil and cranberry juice.

Hosseini et al. (33) investigated fish oil supplementation in 42 rheumatoid arthritis patients over 8 weeks, with doses of 2 g/day for the first 4 weeks followed by 3 g/day for the remaining 4 weeks. Significant reductions in CRP (from 5.1 ± 1.4 to 2.8 ± 1.2 mg/dL, *p* = 0.002) and ESR (from 36 ± 9 to 20 ± 7 mm/h, *p* = 0.003) were observed after 8 weeks, alongside clinically significant improvements in joint inflammation.

Other studies also highlighted additional benefits of fish oil supplementation. Proudman et al. (37) found that a unit increase in EPA (1% of total fatty acids) corresponded to a 12% higher likelihood of achieving remission. However, Magaro et al. (42) did not observe significant clinical benefits with PUFA supplementation, and Nordström et al. (43) reported no effects of alpha-linolenic acid (ALA) supplementation on disease activity.

Park et al. (36) used oleic acid (monounsaturated fatty acids, MUFA) as a control group in a study comparing the effects of n-3 PUFA supplementation on RA outcomes. While omega-3 PUFA provided measurable benefits, MUFA did not significantly influence disease activity markers. Dawczynski et al. (31) reported cardioprotective effects of PUFA after an 8-month trial with 45 participants, suggesting that the inclusion of MUFA in combination with PUFA supplementation could influence lipid profiles and cardiovascular risk factors in RA patients.

Additional studies explored dietary interventions combining MUFAs and PUFAs. Goat and sheep cheese were identified as rich sources of PUFA, with long-term consumption potentially reducing atherosclerosis risk by modulating blood lipids and cardiovascular health markers (75).

Aryaeian et al. (26) conducted a randomized, double-blind, placebo-controlled trial with 70 patients with active RA to evaluate the effects of ginger supplementation. Participants received 1,500 mg of ginger powder daily for 12 weeks, resulting in a significant reduction in DAS28-ESR scores (*p* = 0.001). Gene expression analysis revealed increased FoxP3 (*p* < 0.05), indicative of enhanced regulatory T cell function, alongside reduced expression of T-bet and RORγt (*p* < 0.05), suggesting decreased pro-inflammatory activity of Th1 and Th17 cells.

Ghavipour et al. (44) explored pomegranate extract supplementation, finding significant reductions in DAS28 scores after 8 weeks. This improvement was attributed to decreases in swollen and tender joint counts, pain intensity, and ESR levels. Similarly, vitamin D supplementation showed potential benefits in RA. Soubrier et al. (46) reported reduced HAQ scores and significant improvements in ESR and CRP levels after 6 months, while Gopinath et al. (45) observed greater pain relief in the vitamin D group compared to controls.

N-acetylcysteine (NAC) supplementation has also been studied for its potential effects on RA. Esalatmanesh et al. (47) reported significant reductions in disease activity, including morning stiffness and DAS28 scores, along with improvements in inflammatory biomarkers such as nitric oxide (NO), ESR, malondialdehyde (MDA), high-sensitivity СRP, and glutathione peroxidase (GPx) after a 3-month trial with 74 participants. Positive effects on blood lipids, including lower HDL-C and fasting blood sugar levels, were also noted.

Quercetin, a potent antioxidant with anti-inflammatory properties, was tested in a 16-week trial involving 32 patients. However, no significant changes in disease activity or inflammatory biomarkers, such as cytokines and CRP, were observed compared to lipoic acid and placebo groups (48).

Finally, LD-1227, a patented marine extract combining fish-derived peptides, lipoproteins, and DNA, was evaluated by Lorenzetti et al. (49). In a 12-week study with 40 patients, the LD-1227 group showed an 81.0% ACR20 response compared to 44% in the n-3 PUFA group. Improvements were also noted in VAS scores, HAQ scores, morning stiffness, and tender points, alongside reductions in inflammatory biomarkers and gene expression.

#### 3.2.3 Probiotics, prebiotics, and synbiotics

Alavi et al. (50) conducted a 6-month double-blind randomized placebo-controlled trial involving 69 RA patients to evaluate the effects of a dietary plant-derived polysaccharide (dPP) supplement. The active compound (AC) group (*n* = 33) showed a 12% reduction in agalactosylated (G0F) glycans (*p* = 0.03), while the placebo group (*n* = 36) exhibited an 11% reduction in fully digalactosylated (G2) glycans (*p* = 0.03). Despite these glycan changes, the AC group showed no significant clinical improvements in DAS28 scores, while the placebo group had a slight decrease (difference = 0.63; 95% CI 0.17, 1.10; *p* = 0.009).

Similarly, Hatakka et al. (52) and Maria de Los Angeles et al. (54) found no statistical differences in DAS28, HAQ, or biochemical parameters with probiotic use. In contrast, Cannarella et al. (51) reported that probiotic consumption for 60 days led to significant decreases in TNF-α and IL-6, along with improved antioxidant capacity. However, the probiotics did not significantly affect the DAS-28 score, suggesting that while inflammation and oxidative stress were reduced, overall disease severity remained unchanged.

Zamani et al. (56) found significant improvements in DAS28 scores, serum insulin levels, HOMA-B function, and CRP concentrations after 8 weeks of probiotic intervention, suggesting a beneficial effect on both inflammation and metabolic markers.

In a 60-day study, Mandel et al. (53) enrolled 45 RA patients, randomly assigning them to either the *Bacillus coagulans* group (*n* = 22) or the placebo group (*n* = 22). The probiotic group showed statistically significant improvements in pain scores, patient global assessment, and self-assessed disability compared to placebo. Additionally, CRP levels decreased, and functional abilities (e.g., walking, daily activities) improved.

In the 8-week trial by Vaghef-Mehrabany et al. (55), *Lactobacillus casei* 01 supplementation resulted in a significant decrease in disease activity (DAS28, *p* = 0.039) and an increase in anti-inflammatory cytokine ratios (IL-10/TNF-α, IL-10/IL-12, and IL-10/total Th1; *p* = 0.039, *p* = 0.012, and *p* = 0.014, respectively). By the end of the study, significant differences were observed between the probiotic and placebo groups in IL-10/IL-12 (*p* = 0.038) and IL-10/total Th1 (*p* = 0.006), suggesting an improved inflammatory profile in RA patients.

In the study by Zamani et al. (57), 54 RA patients were randomized to receive either a synbiotic capsule containing *Lactobacillus acidophilus, Lactobacillus casei,* and *Bifidobacterium bifidum* (2 × 10^9^ CFU/g each) plus 800 mg inulin or a placebo for 8 weeks.

Compared with placebo, synbiotic supplementation resulted in a significant improvement in DAS28 (−1.6 ± 0.8 vs. –0.3 ± 0.5, *p* < 0.001) and VAS pain scores (−30.4 ± 18.7 vs. –11.5 ± 15.9, *p* < 0.001), along with reductions in CRP and ESR. Additionally, metabolic markers, including insulin levels (−13.8 ± 26.4 vs. +4.2 ± 28.2 pmol/L, *p* = 0.01), HOMA-IR (*p* = 0.03), and HOMA-B (*p* = 0.01), were significantly improved, and plasma reduced glutathione levels increased (+36.6 ± 63.5 vs. –58.5 ± 154.4 μmol/L, *p* = 0.005). In contrast, Esmaeili et al. (58) conducted a larger 12-week randomized, placebo-controlled trial involving 186 RA patients who received either a daily 1,000 mg synbiotic supplement or a placebo alongside standard methotrexate and prednisolone treatment. Although significant within-group reductions in DAS28, TJC28, and SJC28 were observed, no significant differences were detected between the synbiotic and placebo groups. While CRP levels decreased in the subgroup receiving higher methotrexate doses (15–20 mg/week), ESR remained unchanged. The overall response rate was similar between groups (65.9% in the synbiotic group vs. 65.3% in the placebo group), suggesting that the synbiotic did not provide additional benefits beyond standard pharmacologic treatment. The authors hypothesized that the short intervention period might have limited the potential benefits and recommended extending the treatment duration to 6 months for a more definitive assessment. Table 1 presents studies on dietary interventions and supplementation in RA.

### 3.3 Effect of nutrition and diet in patients with axSpA

Research on the role of diet and nutrition in axSpA is limited. Most available studies focus on specific dietary interventions, such as PUFA supplementation, probiotics, and synbiotics. The limited number of studies underscores the need for further research to draw definitive conclusions about the role of diet and nutrition in axSpA management (69–72).

#### 3.3.1 Supplementation

Sundström et al. (72) conducted a 21-week trial involving 24 patients, comparing high-dose (4.55 g/day) and low-dose (1.95 g/day) PUFA supplementation. Disease activity, functional impairment, ESR, and drug consumption were assessed at baseline and weeks 7, 14, and 21. Eighteen patients completed the study, with the high-dose group showing a significant reduction in BASDAI (*p* = 0.03), while no significant changes were observed in the low-dose group. However, no significant differences were found in drug consumption or functional capacity in either group, nor when comparing the high- and low-dose groups directly. This suggests that higher doses of PUFA may be required to achieve therapeutic effects, but larger controlled trials are needed to confirm these findings.

#### 3.3.2 Probiotics and synbiotics

Jenks et al. (69) and Brophy et al. (71) investigated the effects of probiotic supplementation in axSpA patients but found no statistically or clinically significant differences in disease activity between the probiotic and placebo groups. In Jenks et al. (69), a 12-week randomized controlled trial with 63 patients showed that probiotic supplementation did not significantly improve BASDAI, BASFI, pain, fatigue, or inflammatory markers compared to placebo. Similarly, Brophy et al. (71) conducted an internet-based 12-week randomized trial with 147 patients, where probiotics also failed to improve global well-being, bowel symptoms, or arthritis severity.

In contrast, Ahangari Maleki et al. (70) demonstrated significant immunomodulatory effects of synbiotic supplementation in a 12-week trial involving 48 patients. The study found that synbiotics significantly reduced the proportion of IL-17 expressing CD4^+^ T cells, downregulated IL-17 and IL-23 gene expression, and decreased serum IL-17 and IL-23 levels. Given the well-established role of these cytokines in driving inflammation in axSpA, these findings suggest that synbiotics may influence key inflammatory pathways. However, despite these immunological changes, synbiotic supplementation did not significantly alter BASDAI or ASDAS-CRP compared with placebo, indicating that while synbiotics may modulate immune responses, their impact on clinical disease activity remains uncertain. Table 2 summarizes studies on axSpA.

### 3.4 Effect of nutrition and diet in patients with PsA

This review includes two studies focusing on PUFA supplementation and dietary interventions to assess their impact on disease activity and metabolic parameters in PsA patients. One study compared the effects of PUFA supplementation to olive oil (73). A hypocaloric diet-placebo and a diet-fish intervention were employed in the other trial (74).

#### 3.4.1 Diet

Leite et al. (74) conducted a 12-week randomized controlled trial involving 97 patients with PsA, comparing the effects of a hypocaloric diet combined with either placebo or n-3 PUFA supplementation (diet-fish group) against a control group receiving only a placebo. Both diet groups demonstrated significant improvements in disease activity, with reductions in DAS28-CRP and BASDAI scores, particularly in the diet-placebo group (−0.6 ± 0.9; *p* = 0.004 and −1.39 ± 1.97; *p* = 0.001, respectively). Additionally, a higher proportion of patients in both diet groups achieved minimal disease activity, a key clinically relevant endpoint that underscores the potential of dietary interventions in PsA management beyond weight loss alone. The diet-fish group experienced significant weight loss (−1.79 ± 2.4 kg; *p* = 0.004), as well as reductions in waist circumference (−3.28 ± 3.5 cm; *p* < 0.001) and body fat (−1.2 ± 2.2%; *p* = 0.006). However, despite these body composition changes, there was no direct correlation between weight loss and disease activity improvement. Notably, improvements in dietary quality, particularly a lower Dietary Inflammatory Index and increased intake of fiber, n-3 PUFA, and antioxidant vitamins, appeared to be more relevant factors in disease activity reduction. Each 100-kcal increase in daily intake was associated with a 3.4-fold worsening of DAS28-ESR scores (OR = 0.34; *p* = 0.03), highlighting the potential impact of dietary patterns on inflammatory status.

#### 3.4.2 Supplementation

Kristensen et al. (73) conducted a 24-week randomized, double-blind, placebo-controlled trial involving 145 patients with PsA, comparing the effects of daily supplementation with 3 g of n-3 PUFA against a control group receiving olive oil. The primary outcome focused on cardiac autonomic function, with secondary endpoints including hemodynamic measures and disease activity markers. The results demonstrated significant improvements in autonomic function among PUFA-supplemented patients, as indicated by an increase in RR intervals (*p* = 0.01) and a decrease in heart rate (*p* = 0.01) in per-protocol analyses. These findings suggest a potential cardioprotective effect of n-3 PUFA, potentially reducing cardiovascular disease risk in PsA patients, who are known to have increased cardiovascular morbidity and mortality. However, disease activity markers such as DAS66/68 and CRP remained unchanged after supplementation, suggesting that PUFA did not significantly alter inflammatory activity in this cohort, likely due to the low baseline disease activity among participants. The study also found no significant changes in blood pressure, pulse wave velocity, or central blood pressure, reinforcing that the primary benefit of PUFA in this context may be through autonomic modulation rather than direct anti-inflammatory effects. Table 3 includes studies on PsA.

## 4 Discussion

This systematic review comprehensively evaluated the influence of nutrition and diet on three primary types of chronic arthritis: RA, axSpA, and PsA. A total of 49 articles were included, providing insights into the potential benefits and limitations of various dietary interventions, supplements, probiotics, and synbiotics.

For RA, the findings highlight the potential of PUFAs to reduce disease activity (measured by DAS28), inflammatory biomarkers (CRP, ESR, IL-6), and NSAID use, while also modulating lipid and glucose metabolism (27, 30, 35, 40, 41). Specific supplements, such as ginger (26), pomegranate (44), vitamin D (45, 46), N-acetylcysteine (47), quercetin (48), and LD-1227 (49), demonstrated positive effects on disease activity and inflammation. However, inconsistencies in outcomes were noted across some trials, possibly due to variations in dosages, study durations, and patient populations.

Probiotics and synbiotics showed mixed results. Some studies reported significant improvements in disease activity and biomarkers (50, 56, 58), while others observed no notable changes (52, 54). This highlights a critical theme across the literature: the evidence is frequently inconsistent, and these neutral or negative findings from trials must be carefully weighed against the positive reports when considering clinical implications. For instance, the positive effects on disease activity observed with *Lactobacillus casei* in the Vaghef-Mehrabany et al. (55) trial contrast sharply with the lack of significant changes found with *Lactobacillus rhamnosus* GG in the Hatakka et al. study (52). Differences in bacterial species (e.g., Lactobacillus, Bifidobacterium), specific strains (e.g., *L. casei* vs. *L. rhamnosus* GG), viability (CFU counts), and the presence or absence of prebiotics in synbiotics contribute to diverse immunomodulatory mechanisms and clinical effects, thereby limiting direct comparability and generalizability of findings across this category of interventions. This underscores that ‘probiotics’ cannot be considered a monolithic intervention and that strain-specific effects are a critical factor limiting the generalizability of findings.

Dietary interventions, including the Mediterranean (62, 63), vegan and gluten-free (64, 65), anti-inflammatory (59, 60), peptide (68), and fasting diets (67), were associated with improvements in disease activity, quality of life, cardiovascular risk factors, and inflammatory biomarkers. However, some studies, such as Park et al. (36), observed limited additional effects of n-3 PUFA supplementation in patients with mild and stable disease activity already on antirheumatic medication, potentially due to a ceiling effect.

For axSpA, high-dose n-3 PUFA supplementation demonstrated significant reductions in disease activity, as measured by BASDAI (72). Probiotics did not show significant effects on disease activity or quality of life (69, 71), whereas synbiotics were associated with immunomodulatory effects. For example, Ahangari Maleki et al. (70) showed that synbiotic supplementation reduced IL-17 expressing CD4^+^ T cells and serum levels of IL-17 and IL-23, which are key drivers of inflammation in axSpA. However, it is crucial to note that these promising biological changes did not consistently translate into significant improvements in clinical disease activity scores like BASDAI or ASDAS-CRP. This highlights a common challenge in nutrition research where effects on surrogate markers do not always correspond to direct clinical benefits, underscoring the importance of patient-relevant outcomes.

For PsA, the role of diet and supplementation remains underexplored. PUFA supplementation showed improvements in cardiac autonomic function (increased RR intervals and decreased heart rate), suggesting potential cardiovascular benefits (73). However, disease activity markers such as DAS66/68 and CRP remained unchanged, likely due to the low baseline disease activity among study participants. Special dietary interventions, including hypocaloric and n-3 PUFA-rich diets, were associated with improvements in disease activity, particularly in the diet-placebo group (74). Weight loss was observed in the diet-fish group, but no correlation was found between weight loss and disease activity. Furthermore, a significant proportion of patients across all groups achieved minimal disease activity, further underscoring the potential role of diet in PsA management.

A key strength of this review is the inclusion of 49 RCTs, allowing for a broad comparison of dietary interventions across three types of arthritis. The studies analyzed covered interventions ranging from short-term (8 weeks) to long-term (12 months), providing insights into both immediate and sustained effects. However, several limitations should be noted. Variations in BMI, gender, disease severity, and duration among participants influenced the outcomes, making comparisons across studies challenging. Differences in study design, such as small sample sizes, short durations, and inconsistent reporting of dropout rates, also limited the reliability of some findings (42, 48). Additionally, variability in the assessment of disease activity and inflammatory biomarkers further complicated direct comparisons. While robust data were available for RA, fewer studies investigated dietary interventions in axSpA and PsA, emphasizing the need for further research in these areas. Finally, the exclusion of studies published in languages other than English is an important limitation. This approach may have introduced a language bias, potentially omitted relevant findings, and thus limiting the global generalizability of our conclusions. This was a pragmatic decision to ensure the accuracy of data interpretation, and we recommend that future reviews on this topic incorporate a multilingual search strategy to provide a more comprehensive evidence base. Furthermore, our search was confined to published articles in major databases and did not extend to grey literature or trial registries. This approach carries a potential risk of reporting bias, as studies with neutral or negative findings may be less likely to be published. Consequently, our review may have overlooked some relevant evidence, and this should be considered when interpreting the findings.

While our review provides valuable insights into the role of nutrition across chronic arthritis types, it is important to contextualize the limited number of eligible RCTs [RCTs for axSpA (3 probiotic/synbiotic RCTs, 1 PUFA RCT) and PsA (2 RCTs)]. This scarcity of evidence from RCTs, while reflecting a true publication gap in high-level evidence for these specific conditions, is also a direct consequence of our stringent inclusion criteria, which exclusively focused on RCTs to ensure the highest level of evidence for causality and efficacy. We acknowledge that observational studies, which were excluded by design, could potentially offer valuable signals and insights into the broader role of nutrition in axSpA and PsA management. While outside the scope of this systematic review, such data could be considered in future research to inform the design and prioritization of subsequent high-quality RCTs.

To advance this field and provide clear, evidence-based guidance, future research should prioritize several key areas. First, methodological rigor must be enhanced through trials with larger sample sizes, longer follow-up periods to assess long-term safety and efficacy, and highly standardized protocols for both dietary and supplement interventions to reduce heterogeneity. This includes developing robust methods for monitoring dietary adherence. Second, to improve clinical applicability, future trials must consistently report on patient-relevant outcomes and evaluate them against established thresholds like the minimal clinically important difference. Third, dedicated research is needed to fill evidence gaps, particularly for under-investigated interventions like synbiotics and for specific populations such as patients with axSpA and PsA. Finally, a greater focus is needed on personalized nutrition, exploring strategies tailored to disease subtype, severity, and individual patient characteristics to optimize arthritis management and support practical implementation in clinical care.

## 5 Conclusion

In RA, PUFAs and supplements such as ginger, pomegranate, and vitamin D have shown potential in reducing disease activity (e.g., DAS28) and inflammatory markers (CRP, ESR). In axSpA, high-dose PUFA supplementation demonstrated significant reductions in disease activity (BASDAI), while synbiotics showed notable immunomodulatory effects, such as reduced IL-17 levels. Probiotics, however, did not yield significant improvements in disease activity or quality of life. For PsA, PUFA supplementation showed benefits for cardiac autonomic function (e.g., increased RR intervals and reduced heart rate), though effects on disease activity markers such as DAS66/68 and CRP were limited. Special diets, including n-3 PUFA rich and hypocaloric regimens, improved disease activity measures and supported weight loss, though the correlation between weight loss and disease activity remains unclear. Personalized nutritional strategies tailored to disease type, severity, and patient characteristics are essential for optimizing arthritis management. Therefore, future research must prioritize methodologically rigorous trials with standardized protocols, patient-relevant outcomes, and a focus on long-term safety and efficacy to build a robust evidence base for the role of nutrition in managing chronic inflammatory arthritis.

## Data Availability

The original contributions presented in the study are included in the article/supplementary material, further inquiries can be directed to the corresponding author.
